# Cost-effectiveness of semaglutide 2.4 mg in chronic weight management in Portugal

**DOI:** 10.1186/s13098-024-01338-4

**Published:** 2024-04-30

**Authors:** Luís Silva Miguel, Mariana Soares, Anamaria Olivieri, Filipa Sampaio, Mark Lamotte, Suramya Shukla, Vasco Conde, Paula Freitas, João Costa, Margarida Borges

**Affiliations:** 1 IQVIA, Lisbon, Portugal; 2 IQVIA, Basel, Switzerland; 3 Department of Public Health and Caring Sciences, Uppsala University, Uppsala, Sweden; 4 IQVIA, Zaventem, Belgium; 5 IQVIA, Gurgaon, India; 6 Novo Nordisk, Lisbon, Portugal; 7 Centro Hospitalar Universitário São João (CHUSJ), Porto, Portugal; 8 Laboratório de Farmacologia Clínica e Terapêutica, Faculdade de Medicina, Universidade de Lisboa, Lisboa, Portugal

**Keywords:** Cost-effectiveness analysis, Obesity, Semaglutide 2.4 mg, Portugal

## Abstract

**Background:**

Obesity and overweight are a significant public health concern. Subcutaneous semaglutide 2.4 mg injection is a glucagon-like peptide-1 (GLP-1) analogue approved by the European Medicines Agency as an adjunct to a reduced calorie diet and increased physical activity (diet and exercise, D&E) for the treatment obesity and overweight in the presence of at least one weight related comorbidity. This study aimed to assess the cost-effectiveness of semaglutide 2.4 mg in combination with D&E compared to D&E alone for the Portuguese setting.

**Methods:**

Analysis were conducted using the Core Obesity Model (COM) version 18, a Markov state transition cohort model, to predict the health outcomes and costs of weight related complications based on changes in surrogate endpoints. Efficacy and safety data were sourced from the STEP trials (Body Mass Index, systolic blood pressure and glycemic status) from a cohort of adults aged on average 48 years with obesity (BMI ≥ 30 kg/m2) and ≥ 1 obesity-related comorbidities, over a time horizon of 40 years. Costs were estimated from the perspective of the Portuguese National Health Service. Sensitivity analyses were conducted to test the robustness of results across a range of assumptions.

**Results:**

On a patient level, Semaglutide 2.4 mg in addition to D&E compared to D&E alone, improved QALYs by 0.098 and yielded higher costs by 1,325 EUR over a 40-year time horizon, with an ICER of 13,459 EUR per QALY gained and 100% probability of cost-effectiveness at the given WTP. Semaglutide 2.4 mg remained cost-effective across all different scenarios and sensitivity analysis at a WTP of 20,000 EUR per QALY. Among the subpopulations examined, Semaglutide 2.4 mg yielded ICERs of 18,459 EUR for patients with BMI ≥ 30 kg/m2 and of 22,657 EUR for patients with BMI ≥ 35 kg/m2.

**Conclusions:**

Semaglutide 2.4 mg was cost-effective compared to D&E alone for patients with obesity (BMI ≥ 30 kg/m2) and weight related comorbidities in Portugal, over a 40-year time horizon.

**Supplementary Information:**

The online version contains supplementary material available at 10.1186/s13098-024-01338-4.

## Background

Obesity presents a significant clinical and public health concern, with prevention and management emerging as global priorities [1]. It is characterized by abnormal or excessive fat accumulation, which can impair health and increase the risk of long-term complications [2]. In Portugal, nationwide surveys have revealed a prevalence of obesity ranging from 22 to 29%, while the prevalence of overweight ranges between 35% and 39% [3, 4]. Overweight and obesity pose a significant risk for various chronic diseases, including among others type 2 diabetes (T2D), cardiovascular diseases, asthma, osteoarthritis and different cancers with great impact on health and wellbeing [2, 5].

The impact of obesity extends beyond health, with substantial economic consequences affecting individuals, caregivers, the healthcare system, and society as a whole. In Portugal, people with obesity and overweight contribute to a direct annual cost of 1.2 billion euros, equivalent to 0.6% of the country’s wealth. The diseases most responsible for these healthcare costs include type 2 diabetes, stroke, ischemic heart disease, and chronic kidney disease. Notably, the cost of treating these diseases is 88 times higher than the cost of managing obesity itself, exceeding 13 million euros annually [6].

Preventing and managing obesity and overweight pose significant challenges due to their complex and multifactorial nature, involving genetic, physiological, behavioral, and environmental factors [7], with international guidelines recommending various therapies, including lifestyle and behavioral interventions, pharmacotherapy, and bariatric surgery [8–10]. Despite the potential for significant health improvements, lifestyle interventions such as diet and exercise (D&E) often result in modest weight loss over short periods of time, with many patients facing challenges with metabolic adaptation and difficulties in maintaining D&E practices, which can contribute to weight regain [11].

Recommendations from the Portuguese Society for Obesity Research (Sociedade Portuguesa para o Estudo da Obesidade, SPEO) state that pharmacological treatment for obesity should be considered for people with Body mass index (BMI) ≥ 30 kg/m2 or BMI between 27 kg/m2 and 29.9 kg/m2 and at least one comorbidity who have not achieved at least 5% weight loss between 3 and 6 months through lifestyle interventions [12]. For patients with severe obesity and associated comorbidities, the Directorate-General of Health (Direção Geral da Saúde, DGS) recommends referral to an Obesity Treatment Centre, with bariatric surgery as an option for patients for whom nonsurgical weight reduction measures have failure for at least one year [13]. Reimbursement for effective pharmacotherapy for obesity is limited in Portugal.

Semaglutide (Wegovy®) 2.4 mg injection is a long-acting glucagon-like peptide-1 (GLP-1) analogue, approved by the European Medicines Agency (EMA), that promotes weight loss via slowing down gastric emptying and thereby reducing hunger and increasing satiety [14]. Approximately 5,000 patients enrolled in five phase 3 trials– the Semaglutide Treatment Effect in People with obesity (STEP) program. In STEP 1 trial, patients who received semaglutide 2.4 mg had clinically significant weight loss (at least a 5% reduction in weight from baseline level) compared with placebo (weight reduction: 14.9% vs. 2.4% at week 68; *P* < 0.001) [14], and superior weight loss at 68 weeks when compared with placebo in STEP 2 trial for weight management in patients with overweight or obesity and T2D [15].

This study aimed to assess the cost-effectiveness of semaglutide 2.4 mg in combination with D&E compared to D&E alone for the treatment of adults with obesity (BMI ≥ 30 kg/m2) with one or more weight-related comorbidities, from the perspective of the Portuguese National Health Service (NHS).

## Methods

### Model description

The Core Obesity Model (COM) version 18, a validated Markov-state transition cohort model in Excel, was used to estimate the cost-effectiveness of semaglutide 2.4 mg in combination with D&E compared to D&E alone for the treatment of adults with obesity (BMI ≥ 30 kg/m2) with one or more weight-related comorbidities (Supplementary Figure S1). The model was designed to evaluate the costs and health outcomes associated with the development of obesity-related complications based on risk factors including BMI, lipids, systolic blood pressure (SBP), and glycemic levels/status. Obesity-related complications included in the model were: T2D, myocardial infarction (MI), unstable angina, stroke and transient ischemic attack (TIA), sleep apnea, colon cancer, post-menopausal breast and post-menopausal endometrial cancers, and knee replacement surgery following osteoarthritis. Complications were selected to respond to weight loss having substantial consequences on healthcare resources and costs, patients’ quality of life, and/or life expectancy, based on an unpublished systematic review of the literature [16]. The impact of treatment on comorbidities was assessed by modeling changes in surrogate endpoints that are known to increase the risk of these conditions, such as BMI, SBP, glycemia, and lipids. STEP clinical trials provided data on these surrogate endpoints, while the relationship between surrogate endpoints and actual health outcomes was incorporated into the model using risk equations. These equations explored the association between these risk factors and the incidence of various diseases. In the model, each health state is assigned a cost and a utility decrement for as long as the cohort remains in that particular state. Events such as knee replacement surgery, stroke, TIA, MI, unstable angina, and bariatric surgery incur a one-time cost and lead to a disutility. These event-related costs and utility decrements are applied in the cycle in which the event takes place. A cycle length of 3 months was used in the first year, allowing for more accurate representation of treatment effects and to account for discontinuation due to non-response. Annual cycles were applied after the first year where half-cycle correction was used to estimate occurrence of state transitions in the middle of each cycle. A detailed description of the model and external validation can be found in previous publications [17, 18]. The perspective of the Portuguese National Health Service (NHS) was adopted. Future costs and quality-adjusted life-years (QALYs) were discounted at 4% yearly. Outcomes were modelled over a lifetime horizon corresponding to 40 years. Model outputs included life-years (LYs), QALYs, and total and disaggregated costs (estimated in 2021 EUR). Incremental cost-effectiveness ratios (ICER) were calculated to express the incremental difference in costs and QALYs between semaglutide 2.4 mg and the D&E alternative. The ICER was judged against a willingness-to-pay (WTP) threshold of 20,000 EUR per additional QALY gained, given that there is no established WTP threshold in Portugal. This study adheres to the guidelines in the Consolidated Health Economic Evaluation Reporting Standards (CHEERS) [19].

### Modelled population

The modelled population was based on a subsample of patients (*n* = 1,470) from the total STEP 1 trial population, corresponding to adults aged 48 years on average with BMI ≥ 30 kg/m2 with one or more obesity-related comorbidity eligible for treatment with semaglutide 2.4 mg. The cohort profile is available in Supplementary Table S1.

### Model inputs

#### Treatment effects and parameter progression

Treatment efficacy estimates were sourced from two of the STEP trials. Changes from baseline in BMI, SBP, total cholesterol, HDL, glycemic status, and the proportion of treatment responders (achieving a ≥ 5% weight loss) were sourced from STEP 1 trial [14], at 28- and 68 weeks. In the base case analysis, treatment duration was assumed to last 2 years and estimates from an intention-to-treat analysis were used (treatment policy estimand). Treatment effect based on an analysis of the full sample was applied in cycle 2 of the model (3–6 months) and treatment effect based on analysis of treatment responders achieving ≥ 5% weight loss was applied in cycles 3 and 4 (6–9 and 10–12 months). A stopping rule was applied where treatment was discontinued for patients not achieving the minimum weight loss criteria for treatment response. Non-responders (16.3%) were attributed an efficacy estimate from analysis of the full sample of patients in the D&E arm, assuming all patients continue following a D&E program irrespective of treatment response. Treatment waning effect beyond the STEP 1 trial was sourced from the 104-weeks STEP 5 trial (*n* = 304) [20]. In cycle 5 (year 2), a ratio of change in the risk factor (weight, proportion responders, SBP, and glycemic status) observed in the STEP 5 trial between week 68 and week 104 was computed and applied to the risk factor change observed at week 68 in STEP 1. This adjustment was made based on the efficacy demonstrated by early responders in STEP 5. Starting from cycle 6 (year 3) and each subsequent cycle, the ratio of risk factor change observed in the full analysis set of STEP 5, between week 68 and week 104, was calculated and applied accordingly (Supplementary Table S1). The changes observed at week 68 from STEP 1 trial were assumed to be maintained for total cholesterol and HDL change as assessment of these risk factors was not performed at this time point.

A catch-up rate was applied after treatment cessation to bring the values of the treatment efficacy on the risk factor endpoints back to their baseline values or to a value on their progression with D&E, depending on whether the cohort remains on D&E (Supplementary Table S3). Natural progression beyond this point was assumed for weight to increase with 0.402 kg/year and 0.486 kg/year in males and females respectively (0.463 on average for the cohort) [21] up to a maximum age of 68 years. For glycemic status, a proportion of the cohort with prediabetes status at baseline was assumed to temporarily revert to normal glucose tolerance in cycle 2 only; the maintenance of prediabetes reversal during treatment was also informed by STEP 5 and a catch-up rate was applied post treatment stop.

Treatment discontinuation was assumed for patients not responding to treatment (i.e. *not* achieving weight loss of ≥ 5% in 28 weeks) and applied from cycle 3 for semaglutide 2.4 mg. Non-responders received efficacy estimates from D&E. (Supplementary Table S4).

Bariatric surgery was used as next line therapy post-treatment. The proportion of the cohort eligible for bariatric surgery was determined by the annual incidence rate of bariatric surgery applied during post-treatment. Eligibility was met when the average BMI of the cohort, corresponded or exceeded the BMI threshold outlined in national guidelines. The efficacy of bariatric surgery in the model results in reductions in BMI, SBP, and lipid levels in the corresponding cycles. Efficacy of bariatric surgery on weight loss was derived from the Swedish Obese Subjects Study [22], taking into account the average effect observed with gastric bypass, laparoscopic banding, and gastrectomy procedures. The efficacy of bariatric surgery in reducing SBP and lipid levels was sourced from a prospective study involving patients who underwent gastric bypass surgery in the UK [23] (Supplemental Table S5).

Treatment related adverse events (AEs) were included in the base-case analyses for semaglutide 2.4 mg including severe gastrointestinal events (e.g., nausea, vomiting, diarrhea, etc.) and non-severe hypoglycemia (Supplementary Table S6).

#### Complications

Transition probabilities between health states and the incidence of health events were derived from published risk equations accounting for factors such as physiological parameters (e.g., BMI), medical history, and demographics. Briefly, first-occurring CV events were predicted using the QRisk3 [24]. Recurrent events were predicted using the Framingham Recurrent CHD [25]. The incidence of T2D was predicted using the QDiabetes risk prediction algorithm [26]. The prevalence of sleep apnea was calculated using data from a multicenter cohort, namely the Sleep Heart Health Study [27]. The incidence of knee replacement was predicted using data from the Hospital morbidity database and Statistics Portugal [28] along with baseline risks from a case-control study [29]. The incidence of colon cancer, post-menopausal breast and post-menopausal endometrial cancers were sourced from the International Agency for Research on Cancer [30], and hazards ratios by BMI for colon cancer from Schlesinger et al. [31] and for post-menopausal breast and endometrial cancers from two systematic reviews and meta-analyses [32, 33].

#### Mortality

Sex and age specific all-cause mortality for the general population was sourced from Portuguese life Table [34]. All-cause mortality was adjusted to exclude deaths due to obesity-related complications by subtracting those from all-cause mortality and obtaining non-disease-specific mortality. The non-disease-specific mortality was then adjusted using hazard ratios (HRs) per unit change in BMI, sourced from a study conducted on a large cohort of adults from the UK Clinical Practice Research Datalink (CPRD) database (*N* = 3.6 million) [35], to account for the increased mortality associated with overweight and obesity. Additionally, case fatality rates specific to MI, unstable angina, stroke, knee replacement and bariatric surgery, as well as HRs representing higher mortality rates post-acute coronary syndrome, stroke, and diabetes, observed in the general population, were applied in the model in the cycle in which each event occurred (Supplementary tables S7, S8, S9). These later, are considered under disease-specific mortality.

#### Utilities

Utility values varied by BMI level, sex, age, and the occurrence of comorbid conditions. In the base case analysis, utility valued associated with BMI levels were informed by 36-Item Short Form Survey (SF-36) data collected in STEP 1 trial and mapped onto SF-6D using the Sheffield algorithm with Portuguese population’s preferences [36]. Thereafter, baseline SF-6D scores were linearly regressed against baseline BMI, controlling for age, presence of coronary artery disease, prediabetes, hypertension, and smoking status at baseline in STEP 1. Regression coefficients were used in the model to provide a baseline, complication-free utility dependent on the cohort’s BMI in cycle, age, and sex (Supplementary Tables S10, S11). Event and health state disutilities were sourced from the literature and selected to represent the marginal complication-specific disutility of each complication. These were applied using an additive approach, as the cohort transitioned between comorbidity health states or experienced events, and avoiding double-counting (Supplementary Table 12).

#### Healthcare resource use and cost inputs

The perspective of the Portuguese NHS was taken on costs, and included the cost of the drug, obesity monitoring, bariatric surgery, and costs of complications and ADs. The price of semaglutide 2.4 mg was provided by Novo Nordisk (Supplementary Table S13). Disease monitoring costs were assumed to consist of 8.28 annual health care visits (including 4.5 medical/surgical, 3.61 general practitioner and 0.16 dietitian visits) on average. The cost of D&E was assumed to be zero to the NHS and fully born by patients. Disease monitoring and D&E costs were applied to both treatment arms. Costs related to treatment of complications were sourced from multiple sources including primary health care and specialized care microdata, published literature and publicly available national unit cost data. These costs were applied either as chronic recurring health state costs, or as one-off events costs. Detailed information on the costs of weight related complications is available in Supplementary Table S14.

### Sensitivity analyses

Key model assumptions are described in Supplementary table S15. One-way sensitivity analyses were conducted to investigate the impact of input parameters and assumptions on model outcomes by varying one parameter at a time. Parameters were varied based on reported 95% confidence intervals (CI), when available. For parameters without a 95% CI, a range of ± 25% around the base-case value was used. All values used in the sensitivity analysis are reported in Supplementary table S16.

Scenario analyses were conducted to explore the impact of input and structural assumptions on model results, such as considering disease-specific mortality only, different treatment discontinuation assumptions, longer treatment durations, slower catch-up rates, use of literature-based estimate of BMI-utility (based on Eq. 5D), and using alternative risk equations for modelling the incidence of first and recurrent cardiovascular events (Supplementary Tables S17, S18, S19, S20). In addition, the cost-effectiveness of semaglutide 2.4 mg was examined among subgroup populations, including patients with BMI ≥ 30 and BMI ≥ 35 (Supplementary Tables S22, S23, S24, S25).

Probabilistic uncertainty analysis, using Monte Carlo simulations with 1000 iterations were performed to produce 95% uncertainty intervals (95% UIs) around the cost and effect estimates. In each iteration, inputs were randomly drawn from specified distributions. Uncertainty simulations were presented on a cost-effectiveness plane, representing the joint distribution of costs and QALYs. The probability of semaglutide 2.4 mg being cost-effective against the comparator given different willingness to pay threshold values was represented on a cost-effectiveness acceptability curve (CEAC).

## Results

### Base case analysis

**Fig. 1:**
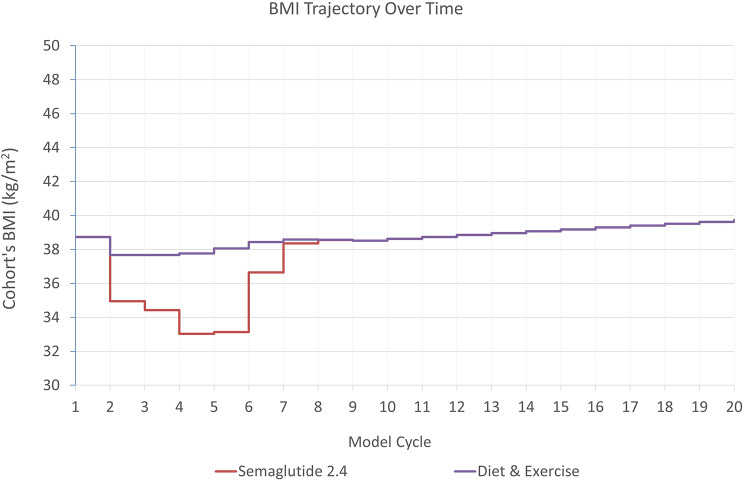
BMI trajectory over time in the base case analysis

**Fig. 2:**
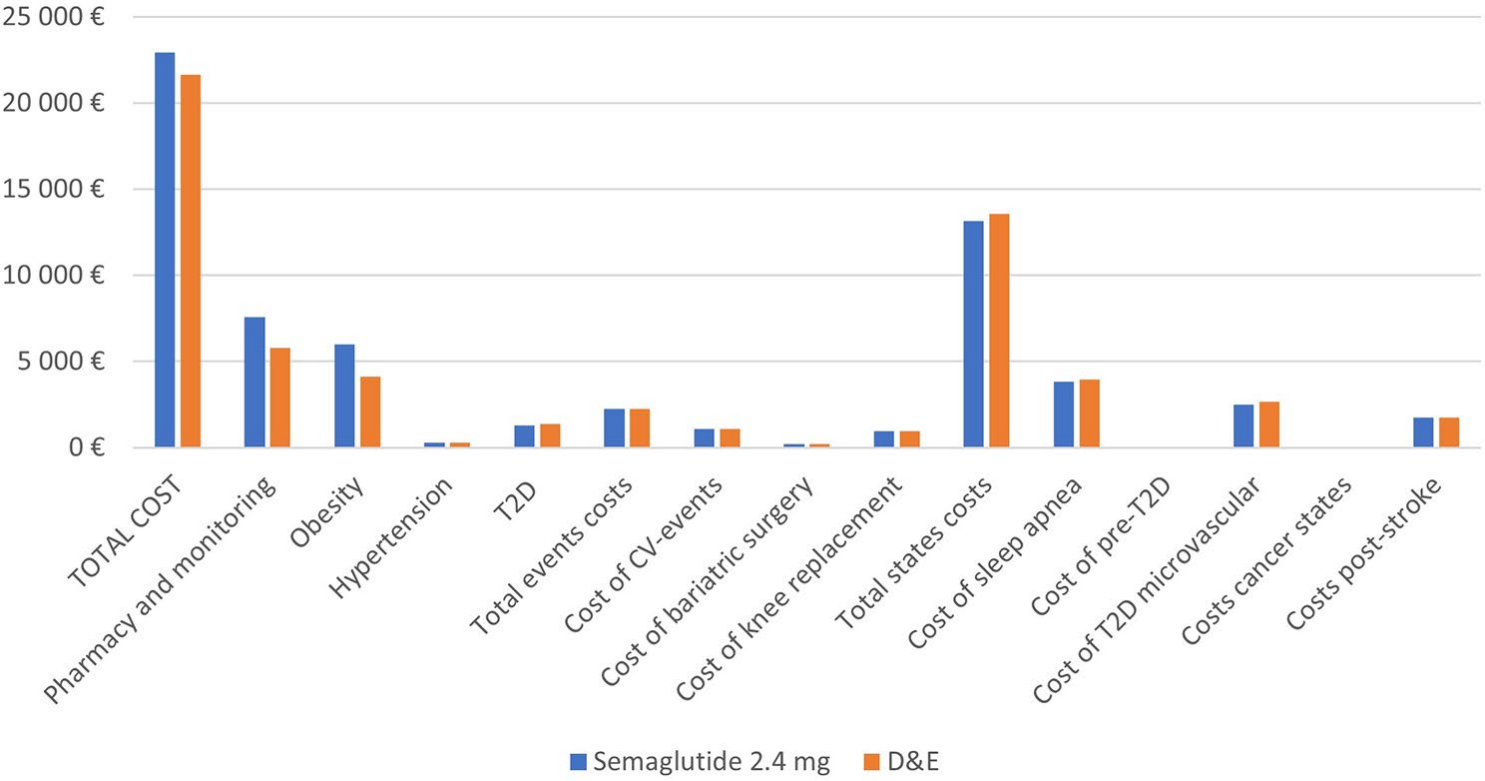
Breakdown of costs Semaglutide 2.4 mg Injection vs. D&E

Estimated mean BMI trajectories over the model period for each treatment arm are presented in Fig. 1. Compared to D&E only, Semaglutide 2.4 mg led to marginally fewer cardiovascular events and knee replacements, and less time with obesity-related complications mostly due to prediabetes reversal and as such a delay in T2D occurrence, and a reduction in sleep apnea prevalence (Table 1). Fewer complications translate into lower costs over the modeling period, with the largest cost offsets related to treatment of complications relating to delayed T2D, reduced sleep apnea, T2D related microvascular complications and certain cancers (Fig. 2 and Supplementary Table S21).

**Table 1:** Breakdown of Clinical Results Semaglutide 2.4 mg Injection vs D&E

	Semaglutide 2.4 mg	D&E	Incremental
**Event Rate per 100 Patient-years**			
CV-events	2.08	2.10	-0.02
Bariatric surgery	0.33	0.33	0
Knee replacement	2.25	2.25	-0.01
**Patient-years in Health State (Undiscounted)**			
No comorbidity + prediabetes reversal	10.51	9.79	0.72
Sleep apnea	11.04	11.25	-0.20
Pre-T2D	7.12	7.38	-0.25
T2D	7.00	7.36	-0.36
Post-ACS	2.38	2.38	0.01
Cancer	1.98	2.02	-0.04
Post-stroke	0.89	0.89	0

ACS– Acute coronary syndrome; CV– Cardiovascular; T2D– Type 2 diabetes; D&E– Diet and exercise

Total costs, LYs, QALYs and ICER estimates over the 40-year modeling period are presented in Table 2. Treatment of obesity with semaglutide 2.4 mg in addition to D&E was cost-effective compared to D&E alone, with an ICER of 13,459 EUR per QALY gained, with incremental 1,325 EUR and 0.098 QALYs.

**Table 2:** Base case cost-effectiveness results for semaglutide 2.4 mg injection vs. diet and exercise

	Semaglutide 2.4 mg	D&E	Incremental vs. D & E
Obesity Pharmacotherapy	1,847	0	1,847
Obesity Monitoring + Diet and Exercise	4162	4138	23
Blood Pressure Treatment	273	272	1
Type 2 Diabetes Pharmacy	1,287	1,377	-90
Complications: Health States	13,140	13,587	-448
Complications: Events	2,248	2,257	-8
**Total costs**	**22,957**	**21,631**	**1,325**
**Total QALYs**	**14.29**	**14.19**	**0.098**
**Total LYs**	**16.23**	**16.15**	**0.078**
**ICER (Cost/QALY gained)**	**13,459**		
**ICER (Cost/LY gained)**	**17,027**		

CEA– Cost-effectiveness analysis; ICER– Incremental cost-effectiveness ratio; LY– Life-years; QALY– Quality adjusted life-years

### Univariate sensitivity analysis

**Fig. 3:**
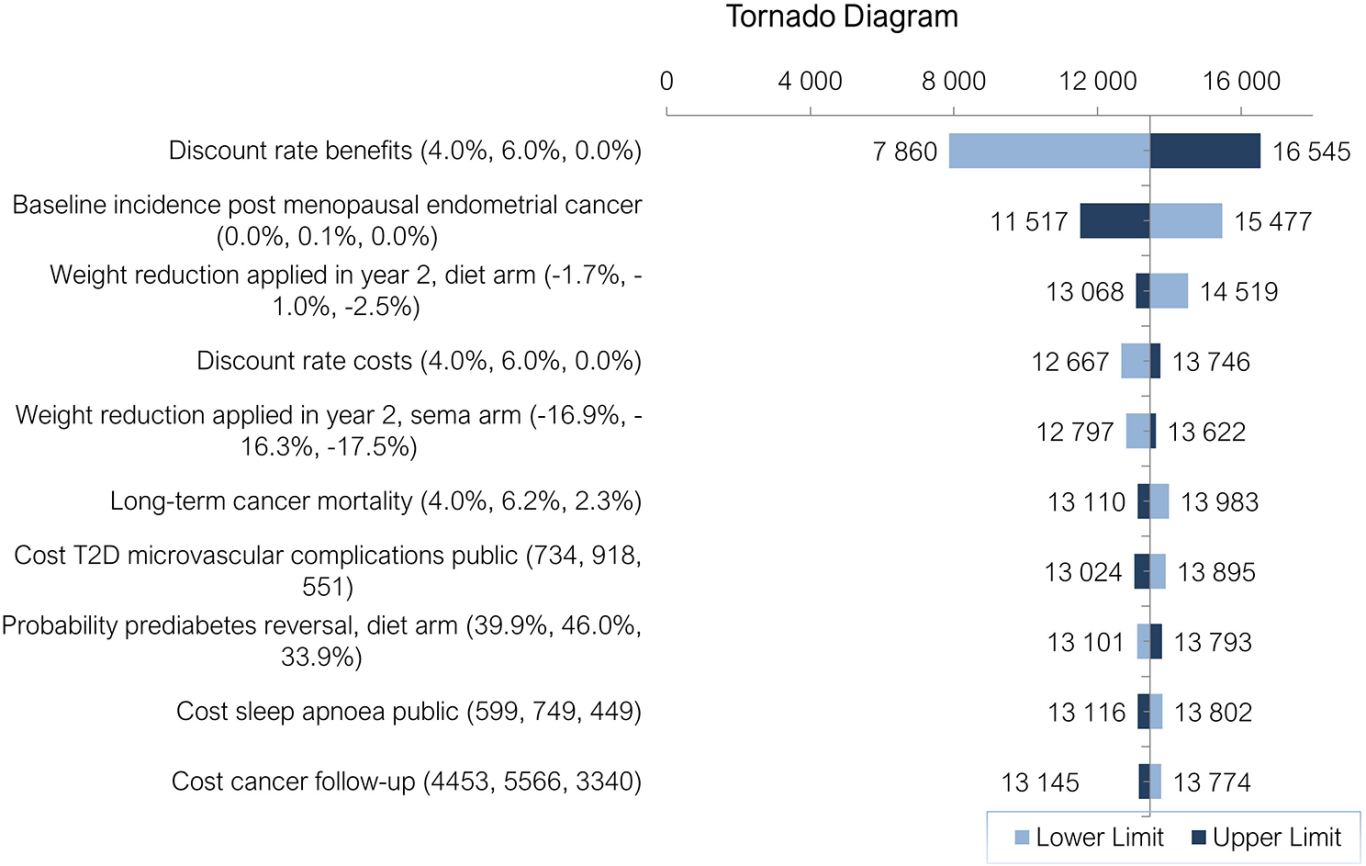
Tornado Diagram Depicting Key Drivers for Cost-Effectiveness of Semaglutide 2.4 mg Injections Compared to D&E. All costs are in EUR €, 2022 values, T2D– type 2 diabetes

The tornado diagram (Fig. 3) shows that ICERs were most sensitivity to variations in the discount rate applied to benefits (0%, 4% and 6%), followed by the baseline incidence of post-menopausal endometrial cancer (for the non-obese Portuguese general population), and weight reductions applied in year 2 of the model on treatment with D&E.

### Scenario analysis

The ICERs remained robust across all scenario analysis, remaining under the WTP threshold of 20,000 EUR, and ranged between 10,797 EUR and 16,006 EUR. The results were most sensitive to the scenario where only disease-specific mortality was considered in the model, resulting in the highest ICER of 16,433 EUR. Longer treatment durations of 3 and 6 years also increased the ICER by 4% and 19% respectively. ICERs decreased when using different treatment estimands (trial product assuming no treatment discontinuation and application of responder efficacy; no stopping rule) and the intention-to-treat sample (treatment policy with non-application of responder efficacy to the semaglutide arm from cycle 3 [stopping rule]). Application of a slower return rate and using a literature-based estimate of BMI-utility further decreased the ICER. Using alternative risk equations for modelling the incidence of first and recurrent cardiovascular events showed little impact on results, in line with the small incremental benefits on cardiovascular outcomes predicted with the model (Table 3).

**Table 3:** Scenario analyses results (in 2021 EUR)

Scenarios		Total costs		Total QALYs		ICER	%-change in ICER vs. BC
		Semaglutide 2.4 mg	D&E	Semaglutide 2.4 mg	D&E		
**Base case**		22,957	21,631	14.292	14.194	13,459	
1	Treatment policy estimand- no stopping rule for Semaglutide 2.4 mg	22,990	21,631	14.297	14.194	13,177	-2%
2	Trial product estimand with stopping rule for Semaglutide 2.4 mg	22,758	21,604	14.295	14.196	11,638	-14%
3	Trial product estimand with No stopping rule for Semaglutide 2.4 mg	22,984	21,604	14.297	14.196	13,679	2%
4	Treatment duration: 3 years	23,532	21,538	14.357	14.215	14,050	4%
5	Treatment duration: 6 years	25,237	21,311	14.492	14.247	16,006	19%
6	Return rate: based on Ara et al. 2012: 33-67-100%	22,889	21,631	14.244	14.127	10,797	-20%
7	Baseline utility: Polynomial/ log function Soltoft et al.	22,957	21,631	14.052	13.935	11,343	-16%
8	Incidence of first CV event in T2D: Qrisk3 Incidence of recurrent CV event in T2D: Framingham Recurrent	23,294	21,983	14.278	14.178	13,080	-3%
9	Disease-mortality only (no BMI-dependent mortality)	25,652	24,418	15.074	14.999	16,433	22%

CV– Cardiovascular; D&E– Diet and exercise; ICER– Incremental cost-effectiveness ratio; QALY– Quality adjusted life-years; SA– Scenario analysis; T2D– Type 2 diabetes

The results for the cost-effectiveness of semaglutide 2.4 mg in other populations are described in the Supplementary Appendix (characteristics of these subpopulations in Supplementary Tables S22, S23 and cost-effectiveness results in Supplementary Tables S24, S25). Notably, semaglutide 2.4 mg was estimated to be cost-effective compared with D&E, in the subgroup of patients with BMI ≥ 30 kg/m2 (ICER = 18,459 EUR). In contrast, the expected ICER for semaglutide 2.4 mg in the subgroup of patients BMI ≥ 35 kg/m2, was 22,657 EUR, slightly above the WTP considered.

### Probabilistic sensitivity analyses

**Fig. 4:**
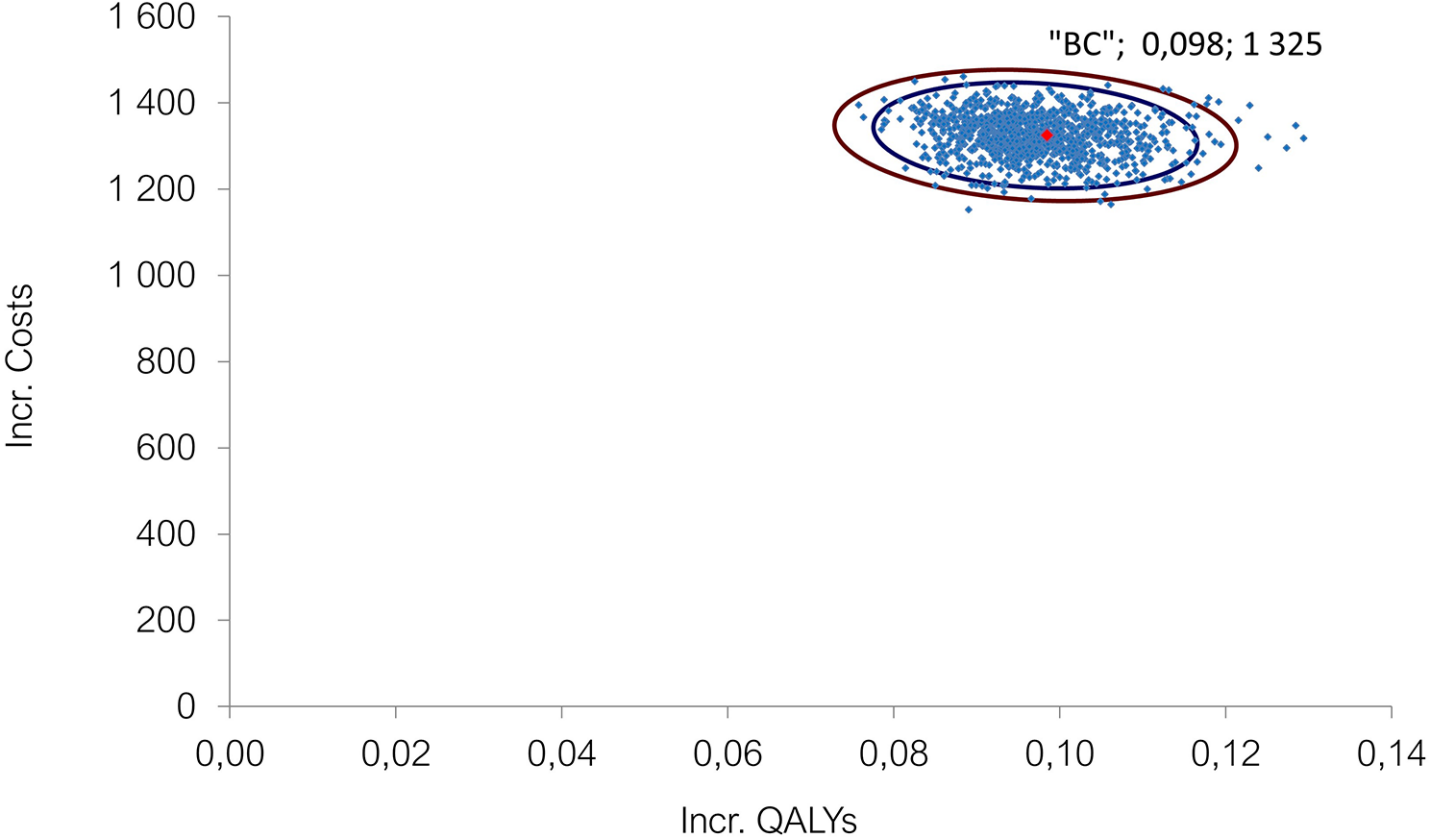
Cost effectiveness plane for semaglutide 2.4 mg injection vs. diet and exercise. Incr.– Incremental; QALY– Quality adjusted life years

**Fig. 5:**
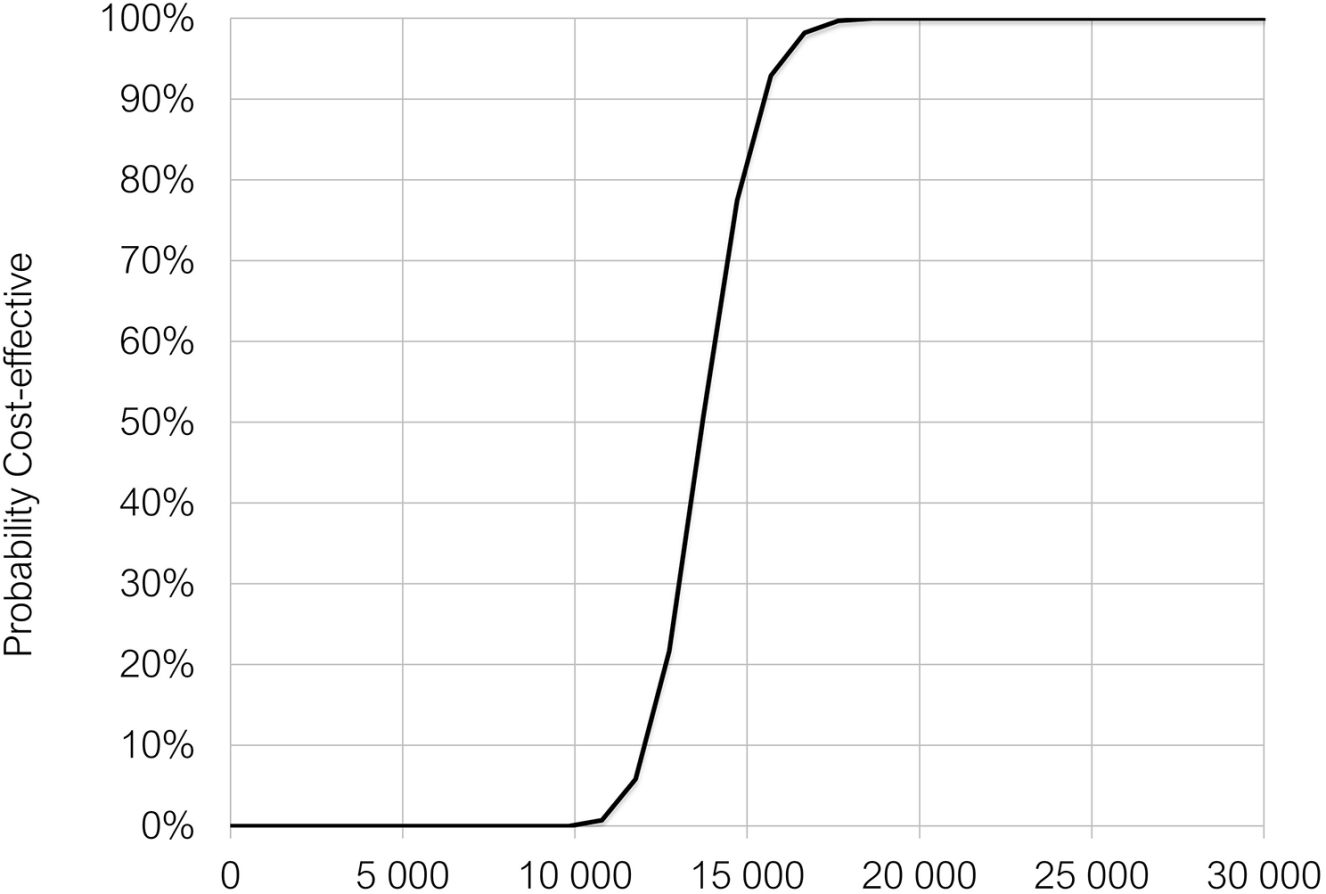
Cost Effectiveness Acceptability Curve for Semaglutide 2.4 mg Injection vs. Diet and Exercise

The cost effectiveness plane depicts the uncertainty around the cost and QALY estimates (Fig. 4). All iterations fell on the upper right quadrant of the plane representing more costs and more QALYs, with semaglutide 2.4 mg having 100% probability of cost effectiveness against D&E alone at the set 20,000 EUR willingness to pay threshold (Fig. 5).

## Discussion

Semaglutide 2.4 mg was estimated to be cost-effective compared with D&E alone, in adults with obesity (BMI ≥ 30 kg/m2) and at least one obesity-related complication from the perspective of the Portuguese NHS.

Efficacy and safety data from STEP 1 and STEP 5 trials used in the cost effectiveness analyses provide robust evidence regarding the effectiveness of semaglutide 2.4 mg in reducing weight, managing blood pressure, controlling lipid levels, and improving glycemic control when compared to relying solely on diet and exercise. The utility data used in these analyses, which inform baseline values and consider age, gender, and BMI, were derived from reported data from STEP 1, and converted to utility weights using country-specific preferences. As a result, the QALY outcomes obtained can be generalized to the broader Portuguese population.

Other studies have estimated the cost effectiveness of semaglutide 2.4 mg in the United Kingdom, Canada, and the United States. Sandhu et al. [37] estimated semaglutide 2.4 mg to be cost-effective against D&E alone with an ICER of £14,827/QALY gained over a lifetime horizon, from the perspective of the NHS and Personal Social Services. Olivieri et al. [38] assessed the cost-effectiveness of weight-management pharmacotherapies approved by Canada Health, from a societal perspective, and have concluded semaglutide 2.4 mg to be the most cost-effective treatment– considering a WTP threshold of CAD 50,000 per QALY– when compared with D&E or orlistat alone, and to dominate other pharmacotherapies, such as NB-32 or liraglutide 3.0 mg. Kim et al. [39] estimated semaglutide 2.4 mg to be cost-effective against other anti-obesity medication (liraglutide 3 mg, phentermine-topiramate, and naltrexone-bupropion), D&E and no treatment, over a lifetime horizon, from a societal perspective, with ICERs varying between $27,113 (vs. no treatment) to $144,296 (vs. phentermine-topiramate), and the ICER for D&E being $22,138.

Although results exhibited variability due to changes in a few parameters and assumptions, results remained robust in all sensitivity and scenario analyses. Additionally, scenario analyses indicate that semaglutide 2.4 mg can be considered cost-effective when responders are assumed to continue treatment for up to six years. Nevertheless, the assumptions made about the continuous use of D&E in non-responders, along with the efficacy estimate applied with a stopping rule in the base case analysis, may have on the one hand resulted in an underestimation of the potential benefits of semaglutide 2.4 mg, however, this may be more representative of its real-world utilization in the Portuguese setting. Additionally, although comprehensive but not exhaustive, the inclusion of complications related to obesity in the model was limited to those with the highest disease and economic burden, whereby the benefits and potential cost savings of weight loss and related complications may go well beyond those considered. The analysis on different subpopulations showed semaglutide 2.4 mg to be cost effective in patients with BMI ≥ 30 kg/m2 but not in patients in BMI ≥ 35 kg/m2. In both analyses, the incremental benefits of semaglutide 2.4 mg against D&E in avoiding cardiovascular events were marginal, which may be explained by these being lower risk populations. Additionally, the average age of these populations were lower than the average age in the base case analysis (46 vs. 48 years in the population with BMI ≥ 30 kg/m2; and 45 vs. 48 years in the population wjth BMI ≥ 35 kg/m2), considering that endometrial and breast cancers were applied in the model post menopause only (with a menopausal average age of 48 years), the benefits accrued with a 2-year weight-loss in this population were smaller compared to a cohort receiving treatment at and post-menopause.

This study has some limitations pertaining to the model structure and parameters. First, there is uncertainty regarding whether a short-term reduction in weight and improvement in other cardiovascular risk factors, depending on the duration of treatment, will lead to a decrease in the occurrence of complications and mortality in individuals who have been obese for an extended period of time. The Swedish Obese Subjects trial [22] provides evidence that supports this. However, it is worth noting that the average weight loss achieved through bariatric surgery in this trial was approximately 23% in the first year, and was maintained at 18% up to 20 years after the initial surgery [40]. While other case-control studies [41] have explored the association between weight reduction and obesity-related complications, longer-term studies and other methodologies are needed to establish a causal link between the weight loss achieved with semaglutide and the reduction or delay of complications over an individual’s lifetime. Such evidence does exist for semaglutide in individuals with T2D, CV disease, or chronic kidney disease (SUSTAIN-6 NCT01720446) [42]. In this context, lower doses of semaglutide (0.5 and 1.0 mg) were significantly associated with a 39% reduction in non-fatal stroke compared to placebo over a median follow-up of 2.1 years, and a non-significant 26% reduction in non-fatal myocardial infarction compared to placebo.

Other limitations pertain to the selection of risk equations. One primary concern is that none of the risk equations employed in the analysis were estimated for the Portuguese population. Therefore, there is uncertainty regarding the extent to which the overall level of risk utilized in the model, as well as the reduction in risks, can be generalized to the Portuguese population. There are, at present, no available risk equations for the Portuguese population. Therefore, the QRisk3, QDiabetes, and UKPDS82 risk equations based on the UK population, may be suitable proxies.

Moreover, efficacy data for semaglutide beyond the duration of the STEP 1 (68 weeks) and STEP 5 (104 weeks) trials are lacking. Real-life estimates are needed to determine the extent of treatment continuation and assess its long-term efficacy. It is only through the availability and use of the product in clinical practice that real-world data on its long-term costs and benefits can be obtained.

Additionally, obesity is associated with several complications, many of which not included in the model. The model has however included those complications that were considered most impactful in terms of disease and economic burden. With this in mind, the full spectrum of potential benefits and cost savings related to weight reduction could not be captured, and current results may be an underestimation of the full impact of this treatment option. For instance, the model did not account for the microvascular complications arising from the progression of T2D and the decline of beta-cell function leading to insulin resistance, hence nor the related changes in costs and quality of life over time. Instead, a single cost and quality of life parameter for T2D were applied throughout the analysis period. This approach may have resulted in an overestimation of T2D costs in the early years and an underestimation in the later years, potentially offsetting each other’s effects.

Finally, there may be some double-counting of mortality cases in the model when both disease-specific mortality and all-cause BMI-dependent mortality are considered. Yet, the inclusion of mortality due to diseases modelled only can be expected to result in an underestimation of mortality [17, 18], and more so in those with higher baseline BMI [43]. Indeed, many population-level studies, including Bhaskaran et al. [35], have found an increased risk of death with increasing weight going beyond the causes currently considered in the COM, such as communicable diseases, liver cirrhosis and liver and kidney cancers, heart failure and atrial fibrillation to name a few. Henceforth, without considering the mortality due to these additional causes, the total life expectancy predicted with the model may lose face validity, especially in populations with higher BMI.

## Conclusions

Semaglutide 2.4 mg was estimated to be a cost-effective treatment alternative to D&E alone for patients with BMI ≥ 30 kg/m2 and at least one weight related comorbidity, in the Portuguese setting, at a WTP of 20,000 EUR per QALY gained. The results were sensitive to the use of different treatment estimands and other input and structural assumptions, with longer use of semaglutide 2.4 mg for 3 and 6 years remaining cost-effective.

### Electronic supplementary material

Below is the link to the electronic supplementary material.

Supplementary Material 1

## Data Availability

There is a restriction applied to the data that support the findings of this study; therefore, they are not publicly available. Data are, however, available from the authors upon contract agreement and with the permission of Novo Nordisk. Please contact the corresponding author.

## Abbreviations

BMI: Body mass index
BP: Blood pressure
CEAC: Cost-effectiveness acceptability curve
CHEERS: Consolidated Health Economic Evaluation Reporting Standards
COM: Core Obesity Model
DGS: Direção Geral da Saúde
D&E: Diet and exercise
EMA: European Medicines Agency
GLP-1: glucagon-like peptide-1
HDL: High density lipoprotein
ICER: Incremental cost-effectiveness ratio
LY: Life years
MI: Myocardial infarction
NHS: National Health Service
SPEO: Sociedade Portuguesa para o Estudo da Obesidade
STEP: Semaglutide Treatment Effect in People with obesity program
SBP: Systolic blood pressure
TIA: Transient ischemic attack
T2D: Type 2 diabetes
WTP: Willingness to pay
