# Supplementary material for: Cost-effectiveness of semaglutide 2.4 mg in chronic weight management in Portugal
Source: Diabetol Metab Syndr. 2024 Apr 30;16:97. doi: 10.1186/s13098-024-01338-4 (PMC11059577; doi:10.1186/s13098-024-01338-4)
Supplement: Supplementary file 1 — Supplementary Material 1 [file 13098_2024_1338_MOESM1_ESM.docx]

**Supplementary appendix**

[**Figure S1**. Core Obesity Model v18 structure. 2](#_Toc138521627)

[**Table S1.** Cohort characteristics at baseline, BMI ≥ 30 kg/m2 and one or more weight-related comorbidity. 3](#_Toc138526325)

[**Table S2.** Percent change in risk factors vs baseline applied in model (Base case). 4](#_Toc138526326)

[**Table S3.** Catch-up rates post-treatment stop. 5](#_Toc138526327)

[**Table S4.** Per-cycle cumulative treatment discontinuation rates. 5](#_Toc138526328)

[**Table S5.** Effect of bariatric surgery (%-change in weight). 6](#_Toc138526329)

[**Table S6.** Rates of Adverse Events Applied in Semaglutide 2.4 mg injection. 6](#_Toc138526330)

[**Table S7.** All-cause mortality, excluding mortality already accounted for in the model, i.e,. non-specific mortality. 7](#_Toc138526331)

[**Table S8.** Hazard Ratios for mortality by BMI level. 8](#_Toc138526332)

[**Table S9.** Disease-specific mortality applied in model. 9](#_Toc138526333)

[**Table S10.** Coefficients applied in the model for females. 9](#_Toc138526334)

[**Table S11.** Coefficients applied in the model for males. 10](#_Toc138526335)

[**Table S12.** Disutilities associated with health states and acute events. 10](#_Toc138526336)

[**Table S13.** Obesity pharmacy costs (all relevant perspectives) (EUR, 2022). 11](#_Toc138526337)

[**Table S14.** Resource use and costs according to the perspective of the Portuguese National Health Service (NHS). 11](#_Toc138526338)

[**Table S15.** Model settings in base case and scenario analyses conducted 22](#_Toc138526339)

[**Table S16.** Input values used in sensitivity analysis. 28](#_Toc138526340)

[**Table S17.** Scenarios: treatment duration beyond 2 years using combined STEP 1 and STEP 5 FAS efficacy. 31](#_Toc138526341)

[**Table S18.** Scenario: trial product estimand using combined STEP 1 and STEP 5 efficacy. 32](#_Toc138526342)

[**Table S19.** BMI and Gender-dependent Baseline Utilities based on EQ-5D responses, used in a scenario analysis. 33](#_Toc138526343)

[**Table S20.** Age-dependent Disutilities based on EQ-5D responses, used in a scenario analysis. 33](#_Toc138526344)

[**Table S21.** Breakdown of Cost Results, € 2022. 34](#_Toc138526345)

[**Table S22:** Cohort characteristics at baseline, BMI ≥ 30 kg/m^2^ (n=1,844). 35](#_Toc138526346)

[**Table S23:** Cohort characteristics at baseline, BMI ≥ 35 kg/m^2^ (n= 1,201). 36](#_Toc138526347)

[**Table S24.** Cost-effectiveness Results for Semaglutide 2.4 mg Injection vs Diet and Exercise, population with BMI ≥30 kg/m^2^ 37](#_Toc138526348)

[**Table S25.** Cost-effectiveness Results for Semaglutide 2.4 mg Injection vs Diet and Exercise, population with BMI ≥35 kg/m^2^ 37](#_Toc138526349)

**Figure S1**. Core Obesity Model v18 structure.

ACS – Acute coronary syndrome; BMI – Body mass index; HbA1c – Haemoglobin A1c; HDL – High density lipoprotein cholesterol; SBP – Systolic blood pressure; T2D - Type 2 diabetes

**Table S1.** Cohort characteristics at baseline, BMI ≥ 30 kg/m2 and one or more weight-related comorbidity.

|  | Mean | Source |
| --- | --- | --- |
| Age (years) | 48.3 | STEP 1 trial |
| BMI (kg/m^2^) | 38.7 | STEP 1 trial |
| Height (m) | 1.67 | STEP 1 trial |
| SBP (mmHg) | 128.0 | STEP 1 trial |
| T-chol (mg/dL) | 196.5 | STEP 1 trial |
| HDL-chol (mg/dL) | 50.8 | STEP 1 trial |
| HbA1c from onset of T2D (%-points) | 7.5 | Clinical expert opinion |
| T2D duration* (years) | 3.0 | Clinical expert opinion |
| Triglycerides (mg/dL) | 146.2 | STEP 1 trial |
| Proportion triglyceride ≥150 mg/dL (%) | 36.5 | STEP 1 trial |
| Smokers (%) | 11.7 | STEP 1 trial |
| Females (%) | 72.9 | STEP 1 trial |
| On lipid-lowering medication (%) | 22.1 | STEP 1 trial |
| On anti-hypertensive medication (%) | 28.8 | STEP 1 trial |
| *Glycaemic status at baseline* |  |  |
| *Normal glucose tolerance (%)* | 45.7 | STEP 1 trial |
| *Prediabetes^†^ (%)* | 52.4 | STEP 1 trial |
| *T2D (%)* | 1.8 | STEP 1 trial |
| History of CVD at baseline^±^ (%) | 2.8 | Data on file: Clinical trial report |

HbA1c - Haemoglobin A1c; T-chol – Total cholesterol; HDL-chol – High density lipoprotein cholesterol; SBP – Systolic blood pressure; T2D – Type 2 diabetes mellitus; †defined as HbA1c of 42 to 47 mmol/mol (6.0 to 6.4%) or FPG of 5.5 to 6.9 mmol/l [1]; ±coronary artery disorders, including coronary artery disease, angina pectoris, myocardial infarction, acute myocardial infarction, myocardial ischemia, arteriosclerosis coronary artery, acute coronary syndrome, angina unstable, coronary artery stenosis, microvascular coronary artery disease, arteriospasm coronary

**Table S2.** Percent change in risk factors vs baseline applied in model (Base case).

| Model cycle | Model year | Semaglutide 2.4 mg injection – responder (base case) | | Diet & exercise full sample (base case) | | | | |
| --- | --- | --- | --- | --- | --- | --- | --- | --- |
|  |  | SE | SE | Mean | SE | | | |
| Base case: treatment policy estimand using combined STEP 1 and STEP 5 efficacy | | | | | |  |  |  |
| Weight | | | | | |  |  |  |
| Cycle 1 | Year 1 | 0 | 0 | 0 | 0 | | | |
| Cycle 2 | Year 1 | -11.12** | 0.17 | -2.74 | 0.24 | | | |
| Cycle 3 | Year 1 | -12.73 | 0.30 | -2.74 | 0.40 | | | |
| Cycle 4 | Year 1 | -17.11 | 0.30 | -2.49 | 0.40 | | | |
| Cycle 5 | Year 2 | -16.93 | 0.30 | -1.75 | 0.40 | | | |
| SBP | | | | | | | | |
| Cycle 1 | Year 1 | 0 | 0 | 0 | 0 | | | |
| Cycle 2 | Year 1 | -5.49** | 0.4 | -0.19 | 0.54 | | | |
| Cycle 3 | Year 1 | -6.39 | 0.4 | -0.19 | 0.54 | | | |
| Cycle 4 | Year 1 | -7.32 | 0.4 | -1.14 | 0.54 | | | |
| Cycle 5 | Year 2 | -7.32 | 0.4 | -1.00 | 0.54 | | | |
| Total cholesterol | | | | | | | |  |
| Cycle 1 | Year 1 | 0.00 | 0.14 | 0.00 | 0.00 | | | |
| Cycle 2 | Year 1 | -14.05** | 0.07 | 0.32 | 0.00 | | | |
| Cycle 3 | Year 1 | -15.57 | 0.07 | 0.32 | 0.00 | | | |
| Cycle 4 | Year 1 | -8.11 | 0.07 | 0.08 | 0.00 | | | |
| Cycle 5 | Year 2 | -8.11 | 0.07 | 0.08 | 0.00 | | | |
| HDL |  |  |  |  |  | | | |
| Cycle 1 | Year 1 | 0.00 | 0 | 0.00 | 0 | | | |
| Cycle 2 | Year 1 | -4.22 | 0.04 | -1.16 | 0.01 | | | |
| Cycle 3 | Year 1 | -4.65 | 0.04 | -1.16 | 0.01 | | | |
| Cycle 4 | Year 1 | 2.49 | 0.02 | 0.56 | 0.01 | | | |
| Cycle 5 | Year 2 | 2.49 | 0.02 | 0.56 | 0.01 | | | |
| Glycemic status | | | | | | | |  |
| Treatment-induced prediabetes reversal | | | | | | | |  |
| Cycle 1 | Year 1 | 0 | 0 | 0 | 0 | | | |
| Cycle 2 | Year 1 | 82.24%** | 1.68% | 39.92% | 3.08% | | | |
| Maintenance of treatment-induced prediabetes reversal using combined STEP 1 and STEP 5 efficacy | | | | | | |  |  |
| Cycle 3 | Year 1 | 82.24%* | 1.55% | 39.92%* | 3.08% | | | |
| Cycle 4 | Year 1 | 82.24%* | 1.55% | 39.92%* | 3.08% | | | |
| Cycle 5 | Year 2 | 81.14% | 1.55% | 24.40% | 3.08% | | | |

Full sample efficacy with semaglutide 2.4 mg injection is always applied in Cycle 2; HDL – High density lipoprotein cholesterol; SE – standard error; SBP – Systolic blood pressure

**Table S3.** Catch-up rates post-treatment stop.

| Cycle after  treatment stop | Weight, SBP, lipids | Reference | Prediabetes reversal | Reference |
| --- | --- | --- | --- | --- |
| **Base case** |  |  |  |  |
| + 1 cycle | 64% | STEP 1 trial | 34% | STEP 1 trial |
| + 2 cycles | 87% | STEP 1 trial | 69% | STEP 1 trial |
| + 3 cycles | 95% | STEP 1 trial | 100% | STEP 1 trial |
| + 4 cycles | 100% | STEP 1 trial |  |  |
| **Scenario** |  |  |  |  |
| + 1 cycle | 33% | [2] | 34% | STEP 1 trial |
| + 2 cycles | 67% | Same rate as in cycle +1 applied cumulatively | 69% | STEP 1 trial |
| + 3 cycles | 100% |  | 100% | STEP 1 trial |

SBP – Systolic blood pressure

**Table S4.** Per-cycle cumulative treatment discontinuation rates.

| Model cycle | Model year | Semaglutide 2.4 mg injection |
| --- | --- | --- |
| Cycle 1 | Year 1 | 0.0% |
| Cycle 2 | Year 1 | 5.8% |
| Cycle 3 | Year 1 | 8.5% |
| Cycle 4 | Year 1 | 10.4% |
| Cycle 5 | Year 2 | 16.6% |
| Cycle 6 | Year 3 | 33.1% |
| Cycle 7+ | Year 4+ | 100% |

Note: Non-responder discontinuation not reported in table but added in the model

**Table S5.** Effect of bariatric surgery (%-change in weight).

|  | Treatment effect | | Reference | Weight (% patients) | |
| --- | --- | --- | --- | --- | --- |
|  | Mean | SE |  | Mean | SE |
| Weight, %-change | -28.3% | 0.1 | Calculated |  |  |
| gastric bypass | -32.0 | 0.1 | [3,4] | 47.5% | 0.1 |
| laparoscopic banding | -20.0 | 0.1 | [3,4] | 0.0% | 0.0 |
| sleeve gastrectomy | -25.0 | 0.1 | [3,4] | 52.5% | 0.1 |
| SBP, mean change (mmHg) | -9.10 | 2.3 | [5] |  |  |
| T-cholesterol, mean change (mg/dl) | -29.69 | 7.2 | [5] |  |  |
| HDL-cholesterol, mean change (mg/dl) | 6.34 | 1.6 | [5] |  |  |
| HbA1c %-point change | -2.15% | 0.0 | [5] |  |  |

HbA1c – Haemoglobin A1c; HDL – High density lipoprotein; SBP – Systolic blood pressure; SE – Standard error; T-cholesterol - Total cholesterol

**Table S6.** Rates of Adverse Events Applied in Semaglutide 2.4 mg injection.

|  | Years of observation | Events | Rate per 100 patient-years | Source |
| --- | --- | --- | --- | --- |
| **Non-severe hypoglycaemia** | | | | |
| Semaglutide 2.4 mg injection | 1856.4 | 15 | 0.8 | Data on file: STEP 1 |
| Placebo | 918.5 | 7 | 0.8 | Data on file: STEP 1 |
| **Severe hypoglycaemia** |  |  |  |  |
| Semaglutide 2.4 mg injection | 1856.4 | 0 | 0 | Data on file: STEP 1 |
| Placebo | 918.5 | 0 | 0 | Data on file: STEP 1 |
| **Severe gastrointestinal** |  |  |  |  |
| Semaglutide 2.4 mg injection | 1856.4 | 91 | 4.9 | Data on file: STEP 1 |
| Placebo | 918.5 | 8 | 0.9 | Data on file: STEP 1 |

**Table S7.** All-cause mortality, excluding mortality already accounted for in the model, i.e,. non-specific mortality.

| **Age in years** | **Males** | **Females** |
| --- | --- | --- |
| Starting age of the cohort: 48 | 0.00277 | 0.00097 |
| 49 | 0.00314 | 0.00104 |
| 50 | 0.00322 | 0.00135 |
| 51 | 0.00367 | 0.00141 |
| 52 | 0.00438 | 0.00160 |
| 53 | 0.00486 | 0.00172 |
| 54 | 0.00513 | 0.00162 |
| 55 | 0.00565 | 0.00201 |
| 56 | 0.00606 | 0.00219 |
| 57 | 0.00652 | 0.00230 |
| 58 | 0.00713 | 0.00219 |
| 59 | 0.00795 | 0.00259 |
| 60 | 0.00874 | 0.00271 |
| 61 | 0.00951 | 0.00313 |
| 62 | 0.01050 | 0.00332 |
| 63 | 0.01102 | 0.00382 |
| 64 | 0.01146 | 0.00397 |
| 65 | 0.01197 | 0.00443 |
| 66 | 0.01287 | 0.00458 |
| 67 | 0.01414 | 0.00526 |
| 68 | 0.01425 | 0.00541 |
| 69 | 0.01552 | 0.00616 |
| 70 | 0.01657 | 0.00671 |
| 71 | 0.01863 | 0.00780 |
| 72 | 0.02019 | 0.00918 |
| 73 | 0.02182 | 0.01034 |
| 74 | 0.02401 | 0.01099 |
| 75 | 0.02641 | 0.01289 |
| 76 | 0.02928 | 0.01497 |
| 77 | 0.03281 | 0.01747 |
| 78 | 0.03577 | 0.01955 |
| 79 | 0.03834 | 0.02217 |
| 80 | 0.04423 | 0.02623 |
| 81 | 0.04924 | 0.03012 |
| 82 | 0.05602 | 0.03452 |
| 83 | 0.06600 | 0.04216 |
| 84 | 0.07972 | 0.05243 |
| 85 | 0.10060 | 0.06909 |
| 86 | 0.11716 | 0.08260 |
| 87 | 0.13511 | 0.09768 |
| 88 | 0.15375 | 0.11360 |
| 89 | 0.17369 | 0.13098 |
| 90 | 0.19697 | 0.15172 |
| 91 | 0.21948 | 0.17209 |
| 92 | 0.24349 | 0.19419 |
| 93 | 0.26893 | 0.21801 |
| 94 | 0.29571 | 0.24350 |
| 95 | 0.32374 | 0.27056 |
| 96 | 0.35285 | 0.29910 |
| 97 | 0.38289 | 0.32895 |
| 98 | 0.41366 | 0.35992 |
| 99 | 0.44493 | 0.39179 |
| 100 | 0.47645 | 0.42429 |

**Table S8.** Hazard Ratios for mortality by BMI level.

| BMI (Kg/m^2^) | HRs | BMI (Kg/m^2^) | HRs | BMI (Kg/m^2^) | HRs | BMI (Kg/m^2^) | HRs |
| --- | --- | --- | --- | --- | --- | --- | --- |
| 15 | 2.25 | 25 | 0.86 | 35 | 1.34 | 45 | 2.42 |
| 16 | 1.99 | 26 | 0.84 | 36 | 1.44 | 46 | 2.51 |
| 17 | 1.76 | 27 | 0.84 | 37 | 1.54 | 47 | 2.60 |
| 18 | 1.56 | 28 | 0.86 | 38 | 1.65 | 48 | 2.67 |
| 19 | 1.39 | 29 | 0.90 | 39 | 1.77 | 49 | 2.74 |
| 20 | 1.24 | 30 | 0.94 | 40 | 1.88 | 50 | 2.79 |
| 21 | 1.12 | 31 | 1.00 | 41 | 1.99 | 51 | 2.83 |
| 22 | 1.02 | 32 | 1.07 | 42 | 2.11 | 52 | 2.83 |
| 23 | 0.95 | 33 | 1.15 | 43 | 2.21 |  |  |
| 24 | 0.89 | 34 | 1.24 | 44 | 2.32 |  |  |

BMI – body mass index; HR – Hazard ratio

**Table S9.** Disease-specific mortality applied in model.

|  | Females | Males | Source |
| --- | --- | --- | --- |
| Case fatality MI | 0.30 | 0.32 | [6] |
| Case fatality angina | 0.30 | 0.32 | Assumed equal to MI |
| Case fatality stroke | 0.25 | 0.17 | [6] |
| Post-ACS | 1.30 | 1.30 | [6] |
| Post-stroke | 2.00 | 2.00 | [7] |
| Colorectal cancer | 0.301 | 0.301 | [8] |
| Breast cancer | 0.041 | N/A | [8] |
| Endometrial cancer | 0.105 | N/A | [8] |
| Cancer | 0.043 | 0.043 | [8] |

MI – myocardial infarction; ACS – Acute coronary syndrome

**Table S10.** Coefficients applied in the model for females.

| Variable name | Description | Coefficient applied  in model |
| --- | --- | --- |
| Intercept | Model Intercept | 1.1928 |
| AGEGR1 | Age (years) | 0.0000 |
| HEARTCIRC1 | Heart or Circulatory Diseases (excl. Hypertension) | -0.0196 |
| HYPER | Hypertension | -0.0039 |
| SMOKE | Smoking Status |  |
| SMOKE | Current Smoker | -0.0077 |
| SMOKE | Previous Smoker | -0.0033 |
| SMOKE | Never Smoked (reference, thus coefficient =0) | 0.0000 |
| BMI | Body Mass Index |  |
| BMI | Linear Effect | -0.0178 |
| BMI2 | Quadratic Effect | 0.0004 |
| BMI3 | Cubic Effect | 0.0000 |
| PRED | Prediabetes Status at Baseline | -0.0008 |

**Table S11.** Coefficients applied in the model for males.

| Variable name | Description | Coefficient applied  in model |
| --- | --- | --- |
| Intercept | Model Intercept | 0.8940 |
| AGEGR1 | Age (years) | 0.0004 |
| HEARTCIRC1 | Heart or Circulatory Diseases (excl. Hypertension) | -0.0303 |
| HYPER | Hypertension | -0.0085 |
| SMOKE | Smoking Status |  |
| SMOKE | Current Smoker | 0.0170 |
| SMOKE | Previous Smoker | 0.0074 |
| SMOKE | Never Smoked (reference, thus coefficient =0) | 0.0000 |
| BMI | Body Mass Index |  |
| BMI | Linear Effect | 0.0044 |
| BMI2 | Quadratic Effect | -0.0002 |
| BMI3 | Cubic Effect | 0.0000 |
| PRED | Prediabetes Status at Baseline | -0.0018 |

**Table S12.** Disutilities associated with health states and acute events.

|  | Mean | SE | Source |
| --- | --- | --- | --- |
| **Disutility applied in health state** | | | |
| T2D  User defined input field (Base case)  Scenario value (Scenario 1-5)  Base case inputs. These are the "default" parameter values stored in the model.  **DSA inputs**  The lower and upper values estimated and stored here are used in the DSA  **PSA inputs**  The random draws for each parameter used in the PSA are estimated and stored here.  Selected scenario.  To change, use the dropdown in the "Cohort Inputs" sheet  Value currently active in the model | -0.029 | 0.007 | [9] |
| Post-ACS | -0.037 | 0.009 | [10] |
| Obstructive sleep apnoea | -0.013 | 0.003 | [9] |
| Cancer | -0.073 | 0.018 | [9] |
| Post-stroke | -0.035 | 0.009 | [10] |
| **Disutility per event** | | | |
| Bariatric Surgery | -0.184 | 0.046 | [11] |
| ACS | -0.129 | 0.032 | [12] |
| Musculoskeletal | -0.023 | 0.006 | [10] |
| Stroke | -0.181 | 0.045 | [12] |
| Transient Ischemic Attacks | -0.033 | 0.008 | [10] |
| Severe GI Events | -0.001 | 0.0002 | [13] |
| Severe Hypoglycaemia | -0.015 | 0.002 | [14] |
| Non-severe Hypoglycaemia | -0.0062 | 0.004 | [14] |

ACS – acute coronary syndrome; SE - Standard error; GI – gastrointestinal; T2D - Type 2 diabetes

**Table S13.** Obesity pharmacy costs (all relevant perspectives) (EUR, 2022).

| Model parameter | Price per Pack (28-days) | Co-payment fee | NHS Cost | Cost to NHS per year | References |
| --- | --- | --- | --- | --- | --- |
| Semaglutide 2.4 mg injection price per day | 228.08 € | 37% | 84.39 € | 1 100.83 € | Novo Nordisk |

**Table S14.** Resource use and costs according to the perspective of the Portuguese National Health Service (NHS).

| Analysis parameter | Costs € | Description & References |
| --- | --- | --- |
| Monitoring costs for obesity, annual | 254 € | For this cost, we only consider resource use regarding medical appointments. This cost estimation required access of microdata from INSEF (Data on file).  This database reports obesity resource use such as the number of medical appointments per patient, with a medical/surgical specialist and with a general practitioner (GP) in a healthcare centre, over 4 weeks, according to several weight ranges (underweight, normal weight, overweight and obese).  The database also reports the resource use of a dietician per patient, according to different weight ranges, as a dummy variable of *Yes/No* use. To this, we assume that the proportion of obese individuals making use of this resource has on average 2 dietician appointments per year (given that data suggests that only 8.06% of obese patients make use of this resource).  Accordingly, the average number of medical/surgical, GP and dietician appointments taken by obese individuals per year, was estimated, according to the INSEF microdata from 2015, amounting to 8.28 appointments on average.  To get the unit cost per appointment type, we resorted to the Portuguese Ordinance *Portaria 207/2017 –* 15^th^ Article [15], which reports the unit cost of 31 € for medical/surgical and GP appointments and 16 € for dietician appointments.  As such, the final cost is estimated as a weighted average between the number of appointments per patient and each unit cost.   \| Appointment type \| Average number of medical appointments per year per obese patient \| Unit cost (€) \| \| --- \| --- \| --- \| \| With medical/surgical specialist \| 4.50 \| 31 € \| \| With GP \| 3.61 \| 31 € \| \| With dietician \| 0.16 \| 16 € \| |
| Diet & exercise | 0 € | This cost is assumed zero, as it is considered to be included in the monitoring costs explained above; also, there is no additional reimbursement for diet and exercise programs at national level. |
| Blood pressure treatment | 55 € | This cost represents the average annual cost of ACE inhibitor treatment of perindopril 5mg +amlodipine 5 mg:  More precisely, the reference price of perindopril and amlodipine of 6,54 € per pack of 30 units [16] was considered. |
| T2D pharmacotherapy | 366 € | This cost estimation required access to microdata from the Regional Health Administration Lisbon and Tagus Valley Information System (*Sistema de Informação da Administração Regional da Saúde Lisboa e Vale do Tejo, SIARS LVT)* (Data on file)*.* SIARS LVT is a repository of administrative and clinical-demographic data from health centre users, which includes all prescriptions to the inhabitants of the region.  From this repository, the authors used a dataset which comprises all the patients with medical diagnosis of T2D or that bought at least two anti-diabetic prescriptions during the year of 2018 and had at least one primary-care medical appointment.   \| Number of eligible patients \| 174 808 \| \| --- \| --- \| \| Male individuals (5) \| 50.4% \| \| Mean Age \| 68.56 \|   Pharmacotherapy costs found in this dataset are described through the Anatomical Therapeutic Chemical (ATC) classification system from the WHO [17]. Accordingly, the annual cost per patient regarding the use of *drugs used in diabetes* (coded with ATC code of A10) was calculated.   \| **ATC code A10: drugs used in diabetes** \| \| \| --- \| --- \| \| Number of eligible patients with T2D \| 174 808 \| \| Average number of packages per patient, per year \| 16.60 \| \| Average annual cost per patient \| 406.88 € \|   To this, the co-payment fee for drugs used in diabetes of 90% supplied by the NHS is applied (considering a public payer perspective), thus amounting to an annual cost of 0.9 * 406.88 = 366.19 €. |
| Non-severe hypoglycaemia^1^ | 0 € | The direct cost for a non-severe hypoglycaemic episode for T2D was assumed zero [18]. |
| Severe hypoglycaemia^2^ | 160 € | The direct cost for a severe hypoglycaemic episode for T2D was calculated as a weighted average between the cost per episode of severe hypoglycaemia and its type of treatment, whether it was hospital-treated, medical/professional-treated or family treated.  The proportions for each treatment type were sourced from Jakubczyk *et al* (2016) [19], which assesses the total annual direct and indirect cost of severe hypoglycaemia events in nine European countries.  Unit costs per type of treatment were sourced as follows:   - Hospital based: inpatient cost of 1 395,35 €, sourced from the BDMH for the year of 2018, accounting for episodes with ICD-10 codes for hypoglycaemia of E16.0, E16.1 or E16.2 as primary diagnosis, and overweight and obesity (ICD-10 code of E66) as additional diagnosis. - Medical Professional based: emergency visit cost of 94.41 €, which is a weighted average between two types of emergency departments (*medico-cirúrgica e polivalente*). Each unit cost is sourced from the Portuguese Ordinance *(Portaria 207/2017)* [15]. - Family treated: assumed zero.   Bearing this, we have:   \|  \| **Proportions (%)** \| **Unit Cost** \| \| --- \| --- \| --- \| \| Hospital Based (hospitalization) \| 9.97% \| 1 395.35 € \| \| Medical professional-treated (emergency visit) \| 22.30% \| 94.41 € \| \| Family Treated \| 67.73% \| 0 € \| |
| Gastrointestinal^3^ adverse event | 95 € | The direct unit cost for severe gastrointestinal events was assumed as the unit cost of an emergency visit (94.41 €) plus the cost per one package of metoclopramide (20 units, 10 mg strength, 1,81 €), which has a co-payment fee of 37% from the NHS [16]. |
| T2D microvascular complications costs (excl. T2D pharmacotherapy) | 734 € | The direct cost of T2D microvascular complications was calculated as the weighted average of inpatient and outpatient costs of neuropathy (including minor and major amputations), nephropathy (including dialysis and kidney transplant) and retinopathy.  Neuropathy and retinopathy costs were based on Gouveia, M, et al. (2010) [20].  Unit costs were updated using:   - 1. Visits and exams: *Portaria 207/2017* [15]*.*   2. Drug costs: Infomed, Infarmed 2022 [16]. For drug classes, we used the weighted average of the price of the prescribed drugs (Moving Annual Total [MAT] sales from IQVIA).   3. Hospitalizations: *Portaria 207/2017* [15] and BDMH of 2018 (Data on file).   Neuropathy:  Outpatient costs with neuropathy = 539 €  Incremental outpatient and inpatient costs with major amputation = 8 398 €  Incremental outpatient and inpatient costs with minor amputation = 4 948 €  Retinopathy:  No inpatient costs were considered. Accordingly, associated costs amount to 622 € per patient, per year.  Nephropathy inpatient and outpatient costs, including dialysis and kidney transplant, were sourced from a Natural evolution model on patients with chronic kidney disease with diabetes, conducted and adapted for the Portuguese reality (Data on file) which estimated a total cost of 39 114 € and approximately 12,63 life-years (LY), thus amounting to 3 096 € per LY.  Finally, the proportions were estimated according to each complication’s prevalence in Portugal.  For the cases of retinopathy and nephropathy, prevalence estimates were sourced from SIARS LVT (Data on file).  For the case of neuropathy, due to the lack of published data for prevalence on amputations, the authors resorted to the DisMod II model, conducted by Barendregt for the WHO [21]. DisMod II extrapolates epidemiologic indicators, resorting to inputs on the total population (sourced from INE 2019 (Data on file)) on incidence rates, case fatality rates, and remission rate of the event (incidence and fatality rates were sourced from the BDMH 2017 (Data on file), and the remission rate was assumed zero).  Bearing this, estimated prevalence, and average unit costs per T2D microvascular complications are as follows:   \|  \| **Weights** \| **Unit Cost** \| \| --- \| --- \| --- \| \| Nephropathy \| 16.21 % \| 3 096.48 € \| \| Neuropathy \|  \|  \| \| Diabetic foot without amputation \| 2.32 % \| 538.73 € \| \| Minor amputation \| 9.31 % \| 5 487.10 € \| \| Major amputation \| 2.67 % \| 8 936.80 € \| \| Retinopathy \|  \|  \| \| Diabetic retinopathy, moderate \| 46.32 % \| 621.50 € \| \| Diabetic retinopathy, severe \| 23.16 % \| |
| Prediabetes | 0 € | Assumed zero (prediabetes costs are included in the obesity monitoring costs). |
| Cancer treatment colon in 1^st^ year | 11 867 € | The direct cost of colon cancer diagnosis and treatment was sourced from the Reference terms for health care systems’ contract, from the Central Administration of the National Health Service [22] for the year of 2022. |
| Cancer treatment breast in 1^st^ year | 10 318 € | The direct cost of breast cancer diagnosis and treatment was sourced from the Reference terms for health care systems’ contract, from the Central Administration of the National Health Service [22] for the year of 2022. |
| Cancer treatment endometrial in 1^st^ year | 12 624 € | The direct cost of endometrial cancer treatment was sourced from the Reference terms for health care systems’ contract, from the Central Administration of the National Health Service [22] for the year of 2022.  Due to the lack of published sources for endometrial cancer, the unit cost for cervical cancer treatment was assumed. |
| Cancer treatment (average of colon, breast, endometrial) in follow-up year | 4 453 € | This cost estimation was calculated as a weighted average.   1. To estimate the proportions per cancer type, prevalence estimates per cancer type were sourced from Globocan [23], for the year of 2020.  \| **Cancers** \| **Prevalence cases 2020, Portugal** \| **Proportions %** \| \| --- \| --- \| --- \| \| Colon \| 14,809 \| 31.9% \| \| Breast \| 27,051 \| 58.3% \| \| Endometrial (corpus uteri) \| 4563 \| 9,8% \|  1. The unit cost per patient per cancer type for the follow-up year was again sourced from Reference terms for health care systems’ contract, from the Central Administration of the National Health Service [22], for the year of 2022, assuming again costs of cervical cancer treatment for the case of endometrial cancer.  \| **Cancers** \| **Cost (follow-up year)** \| \| --- \| --- \| \| Colon \| 5 245 € \| \| Breast \| 4 141 € \| \| Endometrial (corpus uteri) \| 3 729 € \| |
| MI 1^st^ year, excl. acute event cost | 2 147 € | Outpatient resource use with MI was based on Costa, J., *et al.* (2021) [24].  Unit costs were updated using:   1. Visits and exams: *Portaria 207/2017* [15]*.* 2. Drug costs: Infomed, Infarmed 2022 [16]. For drug classes, we used the weighted average of the price of the prescribed drugs (Moving Annual Total [MAT] sales from IQVIA). |
| Unstable angina 1st year, excl. acute event cost | 1 948 € | This cost was assumed equal to the cost above, minus the costs associated with cardiac rehabilitation. |
| Post-acute coronary syndrome (MI or angina, in year following the event) excl. acute event cost | 1 523 € | Outpatient resource use were again based on Costa, J., *et al.* (2021) [24].  Unit costs were updated using:   1. Visits and exams: *Portaria 207/2017* [15]*.* 2. Drug costs: Infomed, Infarmed 2022 [16]. For drug classes, we used the weighted average of the price of the prescribed drugs (Moving Annual Total [MAT] sales from IQVIA). |
| Stroke 1st year, excl. acute event cost | 8 204 € | This cost was calculated was a weighted average of the cost of ischemic stroke and haemorrhagic stroke for the 1st year.   1. Outpatient resource use was based on Costa, J., *et al.* (2021) [24].     Unit costs were updated using:   1. Visits and exams: *Portaria 207/2017* [15]*.* 2. Drug costs: Infomed, Infarmed 2022 [16]. For drug classes, we used the weighted average of the price of the prescribed drugs (Moving Annual Total [MAT] sales from IQVIA).   Bearing this, we have:   - Outpatient costs of ischemic stroke for the 1^st^ year = 8 229 € - Outpatient costs of haemorrhagic stroke for the 1^st^ year = 8 029 €  1. The proportions were estimated according to the number of episodes of each event, sourced from the BDMH 2018 (detailed information can be seen in see Appendix V).  \|  \| **Weights** \| **Unit Cost** \| \| --- \| --- \| --- \| \| Ischemic stroke \| 87.15 % \| 8 229.32 € \| \| Haemorrhagic stroke \| 12.85 % \| 8 029.11 € \| |
| TIA, 1^st^ year, excl. acute event cost | 122 € | Outpatient resource use was based on Costa, J., *et al.* (2021) [24].  Unit costs were updated using:   - 1. Visits and exams: *Portaria 207/2017* [15]*.*   2. Drug costs: Infomed, Infarmed 2022 [16]. For drug classes, we used the weighted average of the price of the prescribed drugs (Moving Annual Total [MAT] sales from IQVIA). |
| Post-stroke (stroke and TIA, in year following the event) | 1 072 € | This cost was calculated was a weighted average between the cost of stroke (ischemic stroke and haemorrhagic stroke) and TIA for the year following the event.   1. Outpatient resource use was based on Costa, J., *et al.* (2021) [24].   Unit costs were updated using:   1. Visits and exams: *Portaria 207/2017* [15]*.* 2. Drug costs: Infomed, Infarmed 2022 [16]. For drug classes, we used the weighted average of the price of the prescribed drugs (Moving Annual Total [MAT] sales from IQVIA). 3. The proportions of stroke and TIA events, used to calculate the weighted average, were sourced from Wolf *et al* (1991) [25].  \|  \| Weights \| Unit Cost \| \| --- \| --- \| --- \| \| TIA \| 21.80 % \| 122.49 € \| \| Ischemic stroke \| 68.15 % \| 1 322.78 € \| \| Haemorrhagic stroke \| 10.05 % \| 1 117.18 € \| |
| Sleep apnoea cost | 599 € | To estimate this cost, the authors assumed the cost of continuous positive airway pressure (CPAP) treatment and associated medical appointments.   1. CPAP daily costs based on *Despacho* n.º 2482/2019 of March 12^th^ [26]. 2. Average number of medical appointments based on expert opinion. Unit cost per medical appointment was sourced from *Portaria 207/2017* [15]. |
| MI non-fatal event cost | 3 359 € | Average cost was estimated through the BDMH of 2018, considering episodes of patients with ≥18 years old, with MI (ICD-10: I21) as main diagnosis, obesity as secondary diagnosis (ICD-10: E66), and a non-fatal outcome.   \| **Average cost** \| **Standard Deviation** \| **Number of episodes** \| **Mean duration of episode (days)** \| \| --- \| --- \| --- \| --- \| \| 3 358.95 € \| 4 000.98 € \| 2051 \| 7.24 \|   Source: BDMH of 2018 and *Portaria 207/2017* [15]*.* |
| MI fatal event cost | 11 323 € | Average cost was estimated through the BDMH of 2018, considering episodes of patients with ≥18 years old, with MI (ICD-10: I21) as main diagnosis, obesity as secondary diagnosis (ICD-10: E66), and a fatal outcome.   \| **Average cost** \| **Standard Deviation** \| **Number of episodes** \| **Mean duration of episode (days)** \| \| --- \| --- \| --- \| --- \| \| 11 323.48 € \| 19 895.19 € \| 86 \| 9.84 \|   Source: BDMH of 2018 and *Portaria 207/2017* [15]*.* |
| Unstable angina non-fatal event cost | 1 311 € | Average cost was estimated through the BDMH of 2018, considering episodes of patients with ≥18 years old, with UA (ICD-10: I20.0) as main diagnosis, obesity as secondary diagnosis (ICD-10: E66), and a non-fatal outcome.   \| **Average cost** \| **Standard Deviation** \| **Number of episodes** \| **Mean duration of episode (days)** \| \| --- \| --- \| --- \| --- \| \| 1 310.99 € \| 741.20 \| 240 \| 2.48 \|   Source: BDMH of 2018 and *Portaria 207/2017* [15]*.* |
| Unstable angina fatal event cost | 9 071 € | For the fatal event cost of unstable angina, no episodes with a fatal outcome were found.  As such, it was estimated by considering episodes of MI as first diagnosis and obesity as additional diagnosis but excluding episodes of percutaneous transluminal coronary angioplasty (PTCA).   \| **Average cost** \| **Standard Deviation** \| **Number of episodes** \| **Mean duration of episode (days)** \| \| --- \| --- \| --- \| --- \| \| 9 071.15 € \| 15 406.11 € \| 63 \| 9.41 \|   Source: BDMH of 2018 and *Portaria 207/2017* [15]*.* |
| Stroke non-fatal event cost | 3 168 € | Average cost was estimated through the BDMH of 2018, considering episodes of patients with ≥18 years old, with stroke as main diagnosis, obesity as secondary diagnosis (ICD-10: E66), and a non-fatal outcome.   \| **Average cost** \| **Standard Deviation** \| **Number of episodes** \| **Mean duration of episode (days)** \| \| --- \| --- \| --- \| --- \| \| 3 168.39 € \| 5 137.78 € \| 2600 \| 14.47 \|   Source: BDMH of 2018 and *Portaria 207/2017* [15]*.* |
| Stroke fatal | 8 364 € | Average cost was estimated through the BDMH of 2018, considering episodes of patients with ≥18 years old, with stroke as main diagnosis, obesity as secondary diagnosis (ICD-10: E66), and a fatal outcome.   \| **Average cost** \| **Standard Deviation** \| **Number of episodes** \| **Mean duration of episode (days)** \| \| --- \| --- \| --- \| --- \| \| 8 364.47 € \| 13 595.26 € \| 292 \| 13.19 \|   Source: BDMH of 2018 and *Portaria 207/2017* [15]*.* |
| TIA event | 1 008 € | Average cost was estimated through the BDMH of 2018, considering episodes of patients with ≥18 years old, with TIA (ICD-10: G459) as main diagnosis and obesity as secondary diagnosis (ICD-10: E66), and a non-fatal outcome.   \| **Average cost** \| **Standard Deviation** \| **Number of episodes** \| **Mean duration of episode (days)** \| \| --- \| --- \| --- \| --- \| \| 1 007.92 € \| 518.56 € \| 253 \| 6.09 \|   Source: BDMH of 2018 and *Portaria 207/2017* [15]*.* |
| Gastric Bypass procedure | 2 252 € | Average cost was estimated through the BDMH of 2018, considering episodes of patients with ≥18 years old, with obesity (ICD-10: E66) as main diagnosis and procedures of gastric bypass.   \| **Average cost** \| **Standard Deviation** \| **Number of episodes** \| **Mean duration of episode (days)** \| \| --- \| --- \| --- \| --- \| \| 2 251.96 € \| 258.75 € \| 1010 \| 3.62 \|   Source: BDMH of 2018, *Portaria 207/2017* [15] and ACSS [27]. |
| Sleeve gastrectomy procedure | 2 279 € | Average cost was estimated through the BDMH of 2018, considering episodes of patients with ≥18 years old, with obesity (ICD-10: E66) as main diagnosis and procedure of sleeve gastrectomy.   \| **Average cost** \| **Standard Deviation** \| **Number of episodes** \| **Mean duration of episode (days)** \| \| --- \| --- \| --- \| --- \| \| 2 278.61 € \| 792.81 € \| 937 \| 3.99 \|   Source: BDMH of 2018, *Portaria 207/2017* [15] and ACSS [27]. |
| Bariatric surgery, preoperative management and post-operative follow-up | 1 548 € | This cost was estimated as a difference between the costs described below, sourced from the reference terms for health care systems’ contract, from ACSS [27] for the year of 2022, and the event costs above.   \| **Unit cost of preoperative management, bariatric surgery, and post-operative follow-up** \| \| \| --- \| --- \| \| Sleeve gastrectomy \| 3 377 € \| \| Gastric Bypass \| 4 295 € \|   Accordingly:   \|  \| Weights \| Unit Cost \| \| --- \| --- \| --- \| \| Sleeve gastrectomy \| 52.42 % \| 1 098.39 € \| \| Gastric Bypass \| 47.58 % \| 2 043.04 € \|   Source: BDMH of 2018, *Portaria 207/2017* [15] and ACSS [27]. |
| Bariatric surgery, complications (leaks) | 1 087 € | The event cost per episode of bariatric surgery complications was calculated using data from the BDMH of 2018, accounting for episodes with ICD-10 procedures codes of bariatric surgery, diagnosis of overweight and obesity, and the Diagnosis Related Group (DRG) code of 252, indicative of “*dysfunction, reaction and/or complication of gastrointestinal device*”.   \| **Average cost** \| **Standard Deviation** \| **Number of episodes** \| **Mean duration of episode (days)** \| \| --- \| --- \| --- \| --- \| \| 1 086.51 € \| 872.22 € \| 404 \| 7.00 \|   Source: BDMH of 2018, *Portaria 207/2017* [15] and ACSS [27].  The percentage of patients with bariatric surgery complications was estimated as 4.26%. |
| Total cost of Bariatric surgery, non-fatal | 3 850 € | This cost is the sum of preoperative management, event costs, additional costs with complications and post-operative follow-up costs described above. |
| Total cost of Bariatric surgery, fatal | 11 549 € | Average cost was estimated through the BDMH of 2018, considering episodes of patients with ≥18 years old, with obesity (ICD-10: E66) as main diagnosis, procedure of bariatric surgery, and a fatal outcome.   \| **Average cost** \| **Standard Deviation** \| **Number of episodes** \| **Mean duration of episode (days)** \| \| --- \| --- \| --- \| --- \| \| 11 549.30 € \| 13 191.22 € \| 2 \| 10.00 \|   Source: BDMH of 2018 and *Portaria 207/2017* [15]*.* |
| Knee replacement, non-fatal event cost | 3 839 € | Average cost was estimated through the BDMH of 2018, considering episodes of patients with ≥18 years old, with obesity as diagnosis (ICD-10: E66), primary or not, procedure of knee replacement surgery, and a non-fatal outcome.   \| **Average cost** \| **Standard Deviation** \| **Number of patients** \| **Number of episodes** \| **Mean duration of episode (days)** \| \| --- \| --- \| --- \| --- \| --- \| \| 3 838.59 € \| 818.69 € \| 1446 \| 1457 \| 7.27 \|   Source: BDMH of 2018 and *Portaria 207/2017* [15]*.* |
| Knee replacement, fatal event cost | 8 584 € | Average cost was estimated through the BDMH of 2018, considering episodes of patients with ≥18 years old, with obesity (ICD-10: E66) as diagnosis, primary or not, procedure of knee replacement, and a fatal outcome.   \| **Average cost** \| **Standard Deviation** \| **Number of patients** \| **Number of episodes** \| **Mean duration of episode (days)** \| \| --- \| --- \| --- \| --- \| --- \| \| 8 584.11 € \| 2 559.90 € \| 7 \| 7 \| 17.00 \|   Source: BDMH of 2018 and *Portaria 207/2017* [15]*.* |

**Table S15.** Model settings in base case and scenario analyses conducted

| Overall settings | Base case (BC) | Scenario | Justification |
| --- | --- | --- | --- |
| Target population | STEP 1 Baseline BMI ≥ 30 kg/m^2^ & ≥ 1 comorbidity | Baseline BMI ≥ 30 kg/m^2^  Baseline BMI ≥ 35 kg/m^2^ | In line with the requested reimbursement population |
| Price semaglutide (€/28-days pack) | €84.39 representing the NHS cost (37%) of a list price of €228.08 per pack | Not conducted | In line with the requested reimbursement price |
| **Analysis settings** | | | |
| Time horizon (years) | 40 | Not conducted | The BC analysis conducted on a cohort with baseline age 48 years and a treatment duration of 2 years. It can be assumed that most costs and quality of life benefits be captured within a 40-years analysis, i.e., by the time the average cohort reaches the age of 88 years. Additionally, most risk equation models informing the prediction of CV risks have 2, 5 or maximum 10-years of follow-up data, thus, applying the prediction of these risk equations over longer periods is associated with high uncertainty. |
| Discount rate costs and benefits | 4% | 0 and 6% | Per pharmacoeconomic guidelines in Portugal [28]. |
| Willingness-to-pay (WTP) (€/QALY) | €20,000 |  | There is no official WTP in Portugal. A WTP of £20,000 per QALY is considered, using an approximate exchange rate £ to € of 1:1 |
| Perspective | Public payer | Not conducted | Per pharmacoeconomic guidelines in Portugal [28]. |
| Include early responders’ analysis (i.e., stop treatment if ≥ 5% weight loss not achieved after 28 weeks) | Yes, for semaglutide 2.4 mg; no for diet and exercise | Not conducted | Patients who do not to achieve a weight loss of 5% or more 3 months after treatment initiation should be discontinued from pharmacological treatment [29]. |
| Estimand | Treatment policy | Trial product | The treatment policy estimand included data from all subjects regardless of premature trial product discontinuation and/or use of rescue medication thus can be considered an intention to treat analysis and was chosen conservatively for the base case analyses.  The trial product estimand estimates the achievable treatment effect without any confounding. The effects of treatment discontinuation are accounted for separately in the model, via non-responder discontinuation and discontinuation due to other reasons. In the proportion of the cohort discontinuing semaglutide 2.4 mg due to non-response the efficacy observed with placebo full analysis set (FAS) in the STEP trial is applied, and the projected results for semaglutide 2.4 mg arm are calculated as the weighted average between efficacy observed in responders and that observed with placebo FAS. |
| Include per-cycle discontinuation before end of fixed treatment period | Yes, for semaglutide 2.4 mg  No, for diet and exercise | No for both semaglutide 2.4 mg and diet and exercise | With semaglutide 2.4 mg, an annual discontinuation rate is applied in addition to discontinuation due to non-response to reflect possible treatment stop due to medication non-adherence, tolerability etc. This is reflective of the discontinuation observed in STEP 1 trial and is only applied to costs in the base case analyses, given the treatment policy estimand is reflective of efficacy observed according to an intention to treat, thus including efficacy post treatment discontinuation.  Diet and exercise are assumed to be continued and no discontinuation is applied. |
| Responders discontinue to | Diet & Exercise | Not conducted | Diet and exercise assumed to be continued lifetime. |
| Maximum duration of treatment (years) | 2 years | 3 and 6 years | Although obesity is a chronic disease and treatment may be expected to continue for many years in patients who respond to and tolerate semaglutide 2.4 mg, clinical efficacy data with semaglutide 2.4 mg was observed up to 104 weeks of treatment. Thus, a two-year treatment duration is chosen in base case analyses, in alignment with the clinical efficacy for semaglutide 2.4 mg substantiated to date.  The impact on cost-effectiveness results for longer treatment durations, up to 3 and up to 6 years was explored in scenario analyses, to reflect the chronic nature of the disease. |
| Assumption about efficacy in years 2+ on treatment | Based on STEP 5 |  | Based on the longest available observed efficacy for semaglutide 2.4 mg in obesity |
| **Adverse events** | | | |
| Include disutility and costs of adverse events | Yes: severe gastrointestinal, severe and non-severe hypoglycaemia | Not conducted | Gastrointestinal adverse events are the most common adverse events observed with semaglutide 2.4 mg (Data on file); the impact on costs and QALYs of severe events was included in base case analyses while moderate and mild events were assumed to resolve at home, without use of significant healthcare resources. Other adverse events observed with treatment, such as hepatobiliary events were only noted in a minority of patients and thus their impact on costs and QALYs can be expected not to influence the conclusions of the CEA conducted. More frequent events occurring with semaglutide 2.4 mg but not with placebo diet, such as headache, and dizziness were considered not to have important, prolonged effect on healthcare costs or quality of life and were thus not included. |
| **Post-treatment assumptions** | | | |
| Fading of treatment effect based on | Return rate |  |  |
| Return rates applied and assumptions | Weight, SBP, lipids: Y1: 64%, Y2: 87%, Y3: 95%, Y4 100%  Drug-induced NGT status: Y1: 34%, Y2: 69% Y3: 100% | Not conducted | Return rates for BMI, SBP, lipids and return to prediabetes status are sourced data observed with semaglutide 2.4 mg in the STEP 1 extension study, and relates to data from patients who have been treated with semaglutide 2.4 mg for weeks 68 (main trial), then discontinued any weight management intervention (including diet). |
| Weight at the end of return period | Return to baseline value | Not conducted |  |
| Natural weight increase after treatment stop | 0.463 kg increase per year [2] until the age of 68, then no change [2,3]. | 0.296 kg increase per year, then 0.296 kg decrease after age of 66. | The annual weight increase in the BC analysis was sourced from a large study with data from the CPRD and the age until weight increased was defined in consultation with a clinical expert. After this age it can be assumed that patients no longer increase in weight and that weight decreases as consequence of muscular mass with possibly no effect on obesity-associated diseases. A scenario was conducted to test the sensitivity of the CEA results to changes in these parameters. |
| **Utilities** | | | |
| Method to derive baseline utility | STEP 1 SF-36 mapped to Portuguese SF-6D | Polynomial/ log function | Base case: SF-36 mapped to SF-6D from STEP 1 trial  **Scenario**: sourced from a published study which controlled for expected confounding effects on the association between weight and quality of life; expected confounding effects in the study were: age, gender, and presence of obesity complications: musculoskeletal, cardiovascular and cancers. |
| **Bariatric surgery** | | | |
| Include bariatric surgery: | Yes | Not conducted | Given, the average BMI before bariatric surgery observed in Portugal was 42 kg/m^2^, bariatric surgery is not expected to be an alternative treatment for the target population, with a baseline BMI of 38 kg/m^2^ and was therefore modelled as a next line therapy. |
| Bariatric surgery criteria - minimum BMI: | 35 kg/m^2^ |  | Based on Portuguese guidelines [30]. |
| Incidence (%) of bariatric surgery per year: | 0.5% |  | Based on number of surgeries performed in Portugal (2013) [31] and total eligible population (BMI≥35 kg/m^2^) [32,33]. |
| **Complications included in the model** | | | |
| Acute coronary syndrome (ACS) | Yes, based on QRisk3 during NGT, Swedish NDR and UKPDS82 for first and recurrent events respectively in T2D | Yes, based on QRisk3 during NGT, QRisk3 and Framingham Recurrent for first and recurrent events respectively in T2D | Based on evidence on the increased risk associated with obesity as well as demonstrated risk reductions associated with weight loss [34–36]. |
| T2D | Yes, based on QDiabetes risk model | Not conducted | Based on evidence on the increased risk associated with obesity as well as demonstrated risk reductions associated with weight loss [34,35,37]. |
| Stroke (including TIA) | Yes, based on same risk models as for ACS | Yes, based on same risk models as for ACS | Based on evidence on the increased risk associated with obesity as well as demonstrated risk reductions associated with weight loss [34–36]. |
| Sleep apnoea | Yes, based on the Sleep heart Study | Not conducted | Based on evidence on the increased risk associated with obesity as well as demonstrated risk reductions associated with weight loss [34,35,38]. |
| Knee replacement | Yes, based on a large (n=911 cases and n=5578 controls) study of Wendelboe et al | Not conducted | Based on evidence on the increased risk associated with obesity as well as demonstrated risk reductions associated with weight loss [34,35,39] |
| Colon cancer | Yes, based on the large study of Adams et al (n~500,000 subjects of whom 3,300 developed colorectal cancer over the study period) | Not conducted | Based on empirical evidence of the increased risk associated with obesity as well as demonstrated risk reductions associated with weight loss [34,35,40]. |
| Post-menopausal endometrial cancer | Yes, based on a systematic literature review reporting incidence of cancer in association with overweight | Not conducted | Based on evidence on the increased risk associated with obesity as well as demonstrated risk reductions associated with weight loss [34,35,41,42]. |
| Post-menopausal breast cancer | Yes, based on a systematic literature review reporting incidence of cancer in association with overweight | Not conducted | Based on evidence on the increased risk associated with obesity as well as demonstrated risk reductions associated with weight loss [34,35,41,42]. |
| Option to apply BMI-adjustment to mortality | Disease mortality & BMI (CPRD) | Not conducted | Other approaches have been shown to underestimate mortality with increasing BMI [43,44]. |

ACS – acute coronary syndrome; BC – base case; BMI – Body mass index; CEA – Cost-effectiveness analysis; CV – Cardiovascular; EOT – End of treatment; FAS – full analysis set; NA – Not applicable; NGT – normal glucose tolerance; NDR – National Diabetes Registry; NICE - National Institute for Health and Care Excellence; OSA - Obstructive sleep apnoea; PRO – patients-reported outcomes; SBP – systolic blood pressure; T2D – Type 2 diabetes; TIA - Transient ischemic attack; US – United states; Y - year

**Table S16.** Input values used in sensitivity analysis.

| **Input** | **Base value** | **Lower value** | **Upper value** |
| --- | --- | --- | --- |
| BMI at baseline | 38.73 | Not varied | Not varied |
| Height at baseline | 1.67 | Not varied | Not varied |
| SBP at baseline | 128.03 | Not varied | Not varied |
| Total cholesterol at baseline | 196.49 | Not varied | Not varied |
| HDL at baseline | 50.76 | Not varied | Not varied |
| HbA1c at baseline | 0.08 | Not varied | Not varied |
| T2D duration | 3.00 | 2.00 | 4.00 |
| Triglyceride >=150 ml/l at baseline | 0.36 | 0.34 | 0.39 |
| Age at menopause | 47.90 | Not varied | Not varied |
| Proportion smokers | 0.12 | 0.10 | 0,13 |
| Proportion females | 0.73 | 0.71 | 0.75 |
| Prediabetes at baseline | 0.52 | Not varied | Not varied |
| T2D at baseline | 0.02 | Not varied | Not varied |
| Natural weight increase | 0.46 | 0.35 | 0.58 |
| Maximum age weight increase | 68.00 | 51.00 | 85.00 |
| Weight reduction sema cycles 2, 3 | -0.13 | -0.13 | -0.12 |
| Weight reduction sema cycle 4 | -0.17 | -0.18 | -0.17 |
| Weight reduction applied in year 2, sema arm | -0.17 | -0.18 | -0.16 |
| Weight reduction applied in year 3, sema arm | -0.17 | -0.17 | -0.16 |
| Weight reduction diet cycles 2, 3 | -0.03 | -0.03 | -0.02 |
| Weight reduction diet cycle 4 | -0.02 | -0.03 | -0.02 |
| Weight reduction applied in year 2, diet arm | -0.02 | -0.03 | -0.01 |
| Weight reduction applied in year 3, diet arm | -0.01 | -0.02 | 0.00 |
| SBP change sema cycles 2, 3 | -6.39 | -7.19 | -5.60 |
| SBP change sema cycle 4 | -7.32 | -6.46 | -8.17 |
| SBP change applied in year 2, sema arm | -7.32 | -8.17 | -6.46 |
| SBP change applied in year 3, sema arm | -7.32 | -8.17 | -6.46 |
| SBP change diet cycles 2, 3 | -0.19 | -1.24 | 0.85 |
| SBP change diet cycle 4 | -1.14 | -0.08 | -2.21 |
| SBP change applied in year 2, diet arm | -1.00 | -2.06 | 0.06 |
| SBP change applied in year 3, diet arm | -0.87 | -1.94 | 0.19 |
| Total cholesterol change sema cycles 2, 3 | -15.57 | -15.84 | -15.30 |
| Total cholesterol change sema cycle 4 | -8.11 | -8.25 | -7.97 |
| Total cholesterol change sema cycle 5 | -8.11 | -8.25 | -7.97 |
| Total cholesterol change sema cycle 6 | -8.11 | -8.25 | -7.97 |
| Total cholesterol change diet cycles 2, 3 | 0.32 | 0.31 | 0.33 |
| Total cholesterol change diet cycle 4 | 0.08 | 0.07 | 0.08 |
| Total cholesterol change diet cycle 5 | 0.08 | 0.07 | 0.08 |
| Total cholesterol change diet cycle 6 | 0.08 | 0.07 | 0.08 |
| HDL change sema cycles 2, 3 | -4.65 | -4.73 | -4.57 |
| HDL change sema cycle 4 | 2.94 | 2.89 | 2.99 |
| HDL change sema cycle 5 | 2.94 | 2.89 | 2.99 |
| HDL change sema cycle 6 | 2.94 | 2.89 | 2.99 |
| HDL change diet cycles 2, 3 | -1.16 | -1.19 | -1.14 |
| HDL change diet cycle 4 | 0.56 | 0.54 | 0.57 |
| HDL change diet cycle 5 | 0.56 | 0.54 | 0.57 |
| HDL change diet cycle 6 | 0.56 | 0.54 | 0.57 |
| A1c change sema cycles 2,3 | 0.00 | Not varied | Not varied |
| A1c change sema cycle 4 | 0.00 | Not varied | Not varied |
| A1c change diet cycles 2,3 | 0.00 | Not varied | Not varied |
| A1c change diet cycle 4 | 0.00 | Not varied | Not varied |
| A1c change diet cycle 5 | 0.00 | Not varied | Not varied |
| A1c change diet cycle 6 | 0.00 | Not varied | Not varied |
| Probability prediabetes reversal, sema arm | 0.82 | 0.79 | 0.86 |
| Probability prediabetes reversal, diet arm | 0.40 | 0.34 | 0.46 |
| Weight reduction discontinuation | 0.00 | Not varied | Not varied |
| Probability non-response sema | 0.16 | 0.14 | 0.19 |
| Probability non-response diet | 0.74 | 0.70 | 0.77 |
| Catch-up time | 1.00 | Not varied | Not varied |
| Proportion MI of CVD | 0.22 | Not varied | Not varied |
| Probability MI is fatal | 0.30 | 0.17 | 0.46 |
| Proportion angina of CVD | 0.55 | Not varied | Not varied |
| Probability angina is fatal | 0.30 | 0.17 | 0.46 |
| Proportion stroke of CVD | 0.24 | Not varied | Not varied |
| Probability stroke is fatal | 0.23 | 0.13 | 0.35 |
| Proportion TIA of stroke | 0.22 | 0.12 | 0.33 |
| Proportion TIA of stroke | 0.22 | 0.12 | 0.33 |
| Rate fatal knee replacement | 0.00 | Not varied | Not varied |
| Rate fatal knee colon cancer | 0.24 | 0.14 | 0.37 |
| Rate fatal knee breast cancer | 0.04 | 0.02 | 0.06 |
| Rate fatal knee endometrial cancer | 0.11 | 0.06 | 0.16 |
| Long-term cancer mortality | 0.04 | 0.02 | 0.06 |
| Baseline incidence colon cancer males | 0.00 | Not varied | Not varied |
| Baseline incidence colon cancer females | 0.00 | 0.00 | 0.01 |
| Rate post menopausal breast cancer | 0.00 | Not varied | Not varied |
| Baseline incidence post menopausal endometrial cancer | 0.00 | Not varied | Not varied |
| Baseline incidence knee replacement under 64 years | 0.00 | Not varied | Not varied |
| Baseline incidence knee replacement over 64 years | 0.00 | Not varied | Not varied |
| Cost monitoring pharmacotherapy public | 254.27 | 190.70 | 317.83 |
| Cost monitoring pharmacotherapy public - first visit | 0.00 | Not varied | Not varied |
| Cost monitoring surgery public | 0.00 | Not varied | Not varied |
| Cost hypertension treatment | 58.05 | 43.54 | 72.56 |
| Cost T2D pharmacy public | 383.85 | 287.89 | 479.81 |
| Cost T2D microvascular complications public | 734.28 | 550.71 | 917.84 |
| Cost prediabetes public | 0.00 | Not varied | Not varied |
| Cost colon cancer public | 11,867.00 | 8,900.25 | 14,833.75 |
| Cost breast cancer public | 10,318.00 | 7,738.50 | 12,897.50 |
| Cost endometrial cancer public | 12,624.00 | 9,468.00 | 15,780.00 |
| Cost cancer follow-up | 4,452.68 | 3,339.51 | 5,565.85 |
| Cost post MI first year public | 2,146.96 | 1,610.22 | 2,683.69 |
| Cost post angina first year public | 1,948.39 | 1,461.29 | 2,435.48 |
| Cost post ACS public | 1,522.77 | 1,142.08 | 1,903.46 |
| Cost post stroke first year public | 8,203.59 | 6,152.70 | 10,254.49 |
| Cost post TIA first year public | 122.49 | 91.87 | 153.11 |
| Cost post stroke public | 1,040.49 | 780.37 | 1,300.62 |
| Cost sleep apnea public | 598.83 | 449.12 | 748.53 |
| Cost non-fatal MI | 3,358.95 | 3,270.60 | 3,447.29 |
| Cost fatal MI | 11,323.48 | 9,178.13 | 13,468.83 |
| Cost non-fatal angina | 1,310.99 | 1,263.14 | 1,358.83 |
| Cost fatal angina | 9,071.15 | 7,130.16 | 11,012.14 |
| Cost non-fatal stroke | 3,168.39 | 3,067.63 | 3,269.15 |
| Cost fatal stroke | 8,364.47 | 7,568.87 | 9,160.07 |
| Cost TIA | 1,007.92 | 975.32 | 1,040.52 |
| Cost non-fatal knee replacement | 3,838.59 | 3,817.14 | 3,860.04 |
| Cost fatal knee replacement | 8,584.11 | 7,616.56 | 9,551.66 |
| Discount rate costs | 0.04 | 0.00 | 0.06 |
| Disutility T2D | -0.03 | -0.04 | -0.02 |
| Disutility post ACS | -0.04 | -0.05 | -0.03 |
| Disutility cancer | -0.07 | -0.09 | -0.05 |
| Disutility stroke | -0.03 | -0.04 | -0.03 |
| Disutility bariatric surgery | -0.18 | -0.23 | -0.14 |
| Disutility MI/angina | -0.13 | -0.16 | -0.10 |
| Disutility knee replacement | -0.02 | -0.03 | -0.02 |
| Disutility stroke | -0.18 | -0.23 | -0.14 |
| Disutility TIA | -0.03 | -0.04 | -0.02 |
| Discount rate benefits | 0.04 | 0.00 | 0.06 |
| Time with osteoarthritis | 3.00 | 0.00 | 5.00 |

ACS – acute coronary syndrome; BMI – body mass index; CVD – cardiovascular disease; HDL – high density lipoprotein; MI – myocardial infarction; SE – standard error; SBP – Systolic blood pressure; TIA – transient ischemic attacks; T2D – type 2 diabetes

**Table S17****.** Scenarios: treatment duration beyond 2 years using combined STEP 1 and STEP 5 FAS efficacy.

| Model cycle | Model year | Semaglutide 2.4 mg injection full set (scenario) | | Diet exercise  – full set (base case) | |
| --- | --- | --- | --- | --- | --- |
|  |  | Mean | SE | Mean | SE |
| Weight | | | |  |  |
| Cycle 6 | Year 3 | -12.36 | 0.28 | -1.23 | 0.40 |
| Cycle 7 | Year 4 | -11.29 | 0.28 | -0.87 | 0.40 |
| Cycle 8 | Year 5 | -10.32 | 0.28 | -0.61 | 0.40 |
| Cycle 9 | Year 6 | -9.43 | 0.28 | -0.43 | 0.40 |
| SBP | | | |  |  |
| **Cycle 6** | **Year 3** | -6.38 | 0.4 | -0.87 | 0.54 |
| **Cycle 7** | **Year 4** | -6.38 | 0.4 | -0.76 | 0.54 |
| **Cycle 8** | **Year 5** | -6.38 | 0.4 | -0.67 | 0.54 |
| **Cycle 9** | **Year 6** | -6.38 | 0.4 | -0.58 | 0.54 |
| **Total cholesterol** | | | | | |
| **Cycle 6** | **Year 3** | -6.78 | 0.05 | 0.08 | 0.00 |
| **Cycle 7** | **Year 4** | -6.78 | 0.05 | 0.08 | 0.00 |
| **Cycle 8** | **Year 5** | -6.78 | 0.05 | 0.08 | 0.00 |
| **Cycle 9** | **Year 6** | -6.78 | 0.05 | 0.08 | 0.00 |
| **HDL** |  |  |  |  |  |
| **Cycle 6** | **Year 3** | 2.49 | 0 | 0.56 | 0 |
| **Cycle 7** | **Year 4** | 2.49 | 0.04 | 0.56 | 0.01 |
| **Cycle 8** | **Year 5** | 2.49 | 0.04 | 0.56 | 0.01 |
| **Cycle 9** | **Year 6** | 2.49 | 0.02 | 0.56 | 0.01 |
| **Glycemic status** | | | | | |
| **Maintenance of treatment-induced prediabetes reversal using combined STEP 1 and STEP 5 efficacy** | | | | | |
| **Cycle 6+** | **Year 3+** | 65.27% | 1.68% | 14.91% | 3.08% |

SE – standard error; SBP – Systolic blood pressure

**Table S18.** Scenario: trial product estimand using combined STEP 1 and STEP 5 efficacy.

| Model cycle | Model year | Semaglutide 2.4 mg injection full set (scenario) | | Diet exercise  – full set (base case) | |
| --- | --- | --- | --- | --- | --- |
|  |  | Mean | SE | Mean | SE |
| Weight | | | |  |  |
| **Cycle 1** | **Year 1** | 0 | 0 | 0 | 0 |
| **Cycle 2** | **Year 1** | -11.90 | 0.17 | -2.79 | 0.24 |
| **Cycle 3** | **Year 1** | -11.90 | 0.28 | -2.79 | 0.40 |
| **Cycle 4** | **Year 1** | -16.59 | 0.28 | -2.56 | 0.40 |
| **Cycle 5** | **Year 2** | -15.86 | 0.28 | -1.83 | 0.40 |
| **SBP** |  |  |  |  |  |
| **Cycle 1** | **Year 1** | 0 | 0 | 0 | 0 |
| **Cycle 2** | **Year 1** | -6.13 | 0.40 | -0.38 | 0.56 |
| **Cycle 3** | **Year 1** | -6.13 | 0.40 | -0.38 | 0.56 |
| **Cycle 4** | **Year 1** | -7.25 | 0.42 | -1.39 | 0.61 |
| **Cycle 5** | **Year 2** | -6.21 | 0.42 | -1.00 | 0.61 |
| **Total cholesterol** | | | | | |
| **Cycle 1** | **Year 1** | 0 | 0 | 0 | 0 |
| **Cycle 2** | **Year 1** | -15.57 | 0.12 | 0.78 | 0.01 |
| **Cycle 3** | **Year 1** | -15.57 | 0.12 | 0.22 | 0.00 |
| **Cycle 4** | **Year 1** | -8.08 | 0.06 | 0.22 | 0.00 |
| **Cycle 5** | **Year 2** | -8.08 | 0.06 | 0.22 | 0.00 |
| **HDL** |  |  |  |  |  |
| **Cycle 1** | **Year 1** | 0 | 0 | 0 | 0 |
| **Cycle 2** | **Year 1** | -4.68 | 0.04 | -1.25 | 0.01 |
| **Cycle 3** | **Year 1** | -4.68 | 0.04 | 0.90 | 0.01 |
| **Cycle 4** | **Year 1** | 2.73 | 0.02 | 0.90 | 0.01 |
| **Cycle 5** | **Year 2** | 2.73 | 0.02 | 0.90 | 0.01 |
| **Glycemic status** | | | | | |
| **Treatment-induced prediabetes reversal** | | | |  |  |
| **Cycle 1** | **Year 1** | 0 | 0 | 0 | 0 |
| **Cycle 2** | **Year 1** | 90.73% | 1.27% | 46.25% | 3.13% |
| **Maintenance of treatment-induced prediabetes reversal using combined STEP 1 and STEP 5 efficacy** | | | | | |
| **Cycle 3** | **Year 1** | 90.73%* | 1.27% | 46.25%* | 3.13% |
| **Cycle 4** | **Year 1** | 90.73%* | 1.27% | 46.25%* | 3.13% |
| **Cycle 5** | **Year 2** | 80.46% | 1.27% | 27.20% | 3.13% |

SE – standard error; SBP – Systolic blood pressure

**Table S19.** BMI and Gender-dependent Baseline Utilities based on EQ-5D responses, used in a scenario analysis.

| BMI (Kg/m^2^) | Utility Females | Utility Males | BMI (Kg/m^2^) | Utility Females | Utility Males |
| --- | --- | --- | --- | --- | --- |
| 30.0 | 0.964 | 0.967 | 40.5 | 0.918 | 0.934 |
| 30.5 | 0.961 | 0.965 | 41.5 | 0.914 | 0.931 |
| 31.0 | 0.959 | 0.963 | 42.5 | 0.911 | 0.929 |
| 31.5 | 0.956 | 0.961 | 43.5 | 0.907 | 0.926 |
| 32.0 | 0.954 | 0.959 | 44.5 | 0.904 | 0.924 |
| 32.4 | 0.952 | 0.959 | 45.5 | 0.901 | 0.921 |
| 33.1 | 0.948 | 0.957 | 46.5 | 0.897 | 0.919 |
| 33.5 | 0.946 | 0.955 | 47.5 | 0.894 | 0.917 |
| 34.0 | 0.943 | 0.955 | 48.5 | 0.891 | 0.915 |
| 34.5 | 0.940 | 0.954 | 49.5 | 0.888 | 0.912 |
| 35.0 | 0.937 | 0.953 | 50.5 | 0.885 | 0.910 |
| 35.5 | 0.934 | 0.952 | 51.5 | 0.882 | 0.908 |
| 36.5 | 0.933 | 0.950 | 52.5 | 0.879 | 0.906 |
| 37.5 | 0.929 | 0.950 | 53.5 | 0.877 | 0.904 |
| 38.5 | 0.925 | 0.948 | 54.5 | 0.874 | 0.902 |
| 39.5 | 0.921 | 0.946 | 55.5 | 0.871 | 0.900 |

**Table S20.** Age-dependent Disutilities based on EQ-5D responses, used in a scenario analysis.

| Age Group | Disutility Applied for Females | Disutility Applied for Males |
| --- | --- | --- |
| 25-34 | 0 | 0 |
| 35-44 | -0.0028 | -0.0213 |
| 45-54 | -0.0081 | -0.0336 |
| 55-64 | -0.043 | -0.0425 |
| 65-74 | -0.0223 | -0.0619 |
| 75+ | -0.0565 | -0.0754 |

**Table S21.** Breakdown of Cost Results, € 2022.

|  | **Semaglutide 2.4 mg** | **D&E** | **Incremental** |
| --- | --- | --- | --- |
| Total cost of pharmacy and monitoring | 7,569 | 5,787 | 1,782 |
| Obesity | 6,008 | 4,138 | 1,870 |
| Hypertension | 273 | 272 | 1 |
| T2D pharmacy | 1,287 | 1,377 | -90 |
| Total cost of events | 2,248 | 2,257 | -8 |
| CV-events | 1,069 | 1,077 | -8 |
| Bariatric surgery | 228 | 227 | 1 |
| Knee replacement | 951 | 952 | -1 |
| Total cost of health state | 13,140 | 13,587 | -448 |
| Sleep apnoea | 3,809 | 3,944 | -135 |
| Pre-T2D | 0 | 0 | 0 |
| T2D microvascular | 2,489 | 2,662 | -173 |
| Post-ACS | 1,728 | 1,730 | -2 |
| Cancer states | 4,645 | 4,781 | -136 |
| Post-stroke | 469 | 471 | -2 |
| **Total cost** | **22,957** | **21,631** | **1,325** |

ACS – Acute coronary syndrome; CV – Cardiovascular; OSA – Obstructive sleep apnoea; T2D – Type 2 diabetes; D&E – Diet and exercise.

**Table S22:** Cohort characteristics at baseline, BMI ≥ 30 kg/m^2^ (n=1,844).

|  | Mean | Source |
| --- | --- | --- |
| Age (years) | 46.1 | STEP 1 trial |
| BMI (kg/m^2^) | 38.4 | STEP 1 trial |
| Height (m) | 1.7 | STEP 1 trial |
| SBP (mmHg) | 126.6 | STEP 1 trial |
| T-chol (mg/dL) | 193.8 | STEP 1 trial |
| HDL-chol (mg/dL) | 50.7 | STEP 1 trial |
| HbA1c from onset of T2D (%-points) | 7.5% | Clinical expert opinion |
| T2D duration* (years) | 3.0 | Clinical expert opinion |
| Triglycerides (mg/dL) | 142.3 | STEP 1 trial |
| Proportion triglyceride ≥150 mg/dL (%) | 33.0% | STEP 1 trial |
| Smokers (%) | 11.6% | STEP 1 trial |
| Females (%) | 74.8% | STEP 1 trial |
| On lipid-lowering medication (%) | 17.9% | STEP 1 trial |
| On anti-hypertensive medication (%) | 22.9% | STEP 1 trial |
| Glycaemic status at baseline |  | STEP 1 trial |
| Normal glucose tolerance (%) | 56.5% | STEP 1 trial |
| Prediabetes^†^ (%) | 41.8% | STEP 1 trial |
| T2D (%) | 1.7% | STEP 1 trial |
| History of CVD at baseline^±^ (%) | 2.8% | Data on file: Clinical trial report |

HbA1c - Haemoglobin A1c; T-chol – Total cholesterol; HDL-chol – High density lipoprotein cholesterol; SBP – Systolic blood pressure; T2D – Type 2 diabetes mellitus; †defined as HbA1c of 42 to 47 mmol/mol (6.0 to 6.4%) or FPG of 5.5 to 6.9 mmol/l [1]; ±coronary artery disorders, including coronary artery disease, angina pectoris, myocardial infarction, acute myocardial infarction, myocardial ischemia, arteriosclerosis coronary artery, acute coronary syndrome, angina unstable, coronary artery stenosis, microvascular coronary artery disease, arteriospasm coronary

**Table S23:** Cohort characteristics at baseline, BMI ≥ 35 kg/m^2^ (n= 1,201).

|  | Mean | Source |
| --- | --- | --- |
| Age (years) | 45.4 | STEP 1 trial |
| BMI (kg/m^2^) | 41.6 | STEP 1 trial |
| Height (m) | 1.7 | STEP 1 trial |
| SBP (mmHg) | 127.7 | STEP 1 trial |
| T-chol (mg/dL) | 191.2 | STEP 1 trial |
| HDL-chol (mg/dL) | 49.1 | STEP 1 trial |
| HbA1c from onset of T2D (%-points) | 7.5% | Clinical expert opinion |
| T2D duration* (years) | 3.0 | Clinical expert opinion |
| Triglycerides (mg/dL) | 143.7 | STEP 1 trial |
| Proportion triglyceride ≥150 mg/dL (%) | 33.3% | STEP 1 trial |
| Smokers (%) | 11.4% | STEP 1 trial |
| Females (%) | 76.2% | STEP 1 trial |
| On lipid-lowering medication (%) | 17.7% | STEP 1 trial |
| On anti-hypertensive medication (%) | 24.8% | STEP 1 trial |
| Glycaemic status at baseline |  | STEP 1 trial |
| Normal glucose tolerance (%) | 52.5% | STEP 1 trial |
| Prediabetes^†^ (%) | 45.5% | STEP 1 trial |
| T2D (%) | 2.0% | STEP 1 trial |
| History of CVD at baseline^±^ (%) | 2.8% | Data on file: Clinical trial report |

HbA1c - Haemoglobin A1c; T-chol – Total cholesterol; HDL-chol – High density lipoprotein cholesterol; SBP – Systolic blood pressure; T2D – Type 2 diabetes mellitus; †defined as HbA1c of 42 to 47 mmol/mol (6.0 to 6.4%) or FPG of 5.5 to 6.9 mm [1] ; ±coronary artery disorders, including coronary artery disease, angina pectoris, myocardial infarction, acute myocardial infarction, myocardial ischemia, arteriosclerosis coronary artery, acute coronary syndrome, angina unstable, coronary artery stenosis, microvascular coronary artery disease, arteriospasm coronary

**Table S24.** Cost-effectiveness Results for Semaglutide 2.4 mg Injection vs Diet and Exercise, population with BMI ≥30 kg/m^2^

|  | Semaglutide 2.4 mg | Diet & exercise | Incremental |
| --- | --- | --- | --- |
| Obesity Pharmacotherapy | 1,831 | 0 | 1,831 |
| Obesity Monitoring + Diet and Exercise | 4,282 | 4,265 | 17 |
| Blood Pressure Treatment | 224 | 223 | 1 |
| Type 2 Diabetes Pharmacy | 1,156 | 1,231 | -75 |
| Complications: Health States | 12,940 | 13,285 | -345 |
| Complications: Events | 2,062 | 2,073 | -11 |
| **Total costs** | 22,494 | 21,077 | 1,417 |
| **Total QALYs** | 14.75 | 14.67 | 0.077 |
| **Total LYs** | 16.71 | 16.66 | 0.054 |
| **ICER (Cost/QALY gained)** |  |  | **18,459** |
| **ICER (Cost/LY gained)** |  |  | 26,390 |

CEA – Cost-effectiveness analysis; ICER – Incremental cost-effectiveness ratio; LY – Life-years; QALY – Quality adjusted life-years

**Table S25.** Cost-effectiveness Results for Semaglutide 2.4 mg Injection vs Diet and Exercise, population with BMI ≥35 kg/m^2^

|  | Semaglutide 2.4 mg | Diet & exercise | Incremental |
| --- | --- | --- | --- |
| Obesity Pharmacotherapy | 1,787 | 0 | 1,787 |
| Obesity Monitoring + Diet and Exercise | 4,162 | 4147.8 | 14 |
| Blood Pressure Treatment | 236 | 235 | 1 |
| Type 2 Diabetes Pharmacy | 1209 | 1283 | -74 |
| Complications: Health States | 15,200 | 15,538 | -337 |
| Complications: Events | 2,113 | 2,124 | -12 |
| **Total costs** | 24,707 | 23,327 | 1,379 |
| **Total QALYs** | 14.21 | 14.15 | 0.061 |
| **Total LYs** | 16.23 | 16.19 | 0.043 |
| **ICER (Cost/QALY gained)** |  |  | **22,657** |
| **ICER (Cost/LY gained)** |  |  | 32,343 |

CEA – Cost-effectiveness analysis; ICER – Incremental cost-effectiveness ratio; LY – Life-years; QALY – Quality adjusted life-years

**References**

1. Overview | Type 2 diabetes: prevention in people at high risk | Guidance | NICE [Internet]. [cited 2023 Jun 23]. Available from: https://www.nice.org.uk/guidance/ph38

2. Ara R, Blake L, Gray L, Hernández M, Crowther M, Dunkley A, et al. What is the clinical effectiveness and cost-effectiveness of using drugs in treating obese patients in primary care? A systematic review. Health Technol Assess. 2012;16:iii–xiv, 1–195.

3. Sjöström L, Lindroos A-K, Peltonen M, Torgerson J, Bouchard C, Carlsson B, et al. Lifestyle, diabetes, and cardiovascular risk factors 10 years after bariatric surgery. N Engl J Med. 2004;351:2683–93.

4. Torgerson JS, Sjöström L. The Swedish Obese Subjects (SOS) study--rationale and results. Int J Obes Relat Metab Disord J Int Assoc Study Obes. 2001;25 Suppl 1:S2-4.

5. Demssie YN, Jawaheer J, Farook S, New JP, Syed AA. Metabolic outcomes 1 year after gastric bypass surgery in obese people with type 2 diabetes. Med Princ Pract Int J Kuwait Univ Heal Sci Cent. 2012;21:125–8.

6. Johansson S, Rosengren A, Young K, Jennings E. Mortality and morbidity trends after the first year in survivors of acute myocardial infarction: a systematic review. BMC Cardiovasc Disord. 2017;17:53.

7. Brammås A, Jakobsson S, Ulvenstam A, Mooe T. Mortality after ischemic stroke in patients with acute myocardial infarction: predictors and trends over time in Sweden. Stroke. 2013;44:3050–5.

8. Cancer-Research-UK. Cancer survival statistics 2010-2011.

9. Gough SC, Kragh N, Ploug UJ, Hammer M. Impact of obesity and type 2 diabetes on health-related quality of life in the general population in England. Diabetes Metab Syndr Obes. 2009;2:179–84.

10. Sullivan PW, Slejko JF, Sculpher MJ, Ghushchyan V. Catalogue of EQ-5D scores for the United Kingdom. Med Decis Mak an Int J Soc Med Decis Mak. 2011;31:800–4.

11. Campbell J, McGarry LA, Shikora SA, Hale BC, Lee JT, Weinstein MC. Cost-effectiveness of laparoscopic gastric banding and bypass for morbid obesity. Am J Manag Care. 2010;16:e174-87.

12. Clarke P, Gray A, Holman R. Estimating utility values for health states of type 2 diabetic patients using the EQ-5D (UKPDS 62). Med Decis Mak an Int J Soc Med Decis Mak. 2002;22:340–9.

13. National Institute for Health and Care Excellence (NICE). Naltrexone–bupropion for managing overweight and obesity - Technology appraisal guidance. [Internet]. 2017. Available from: https://www.nice.org.uk/guidance/ta494

14. Foos V, McEwan P. Conversion of Hypoglycemia Utility Decrements from Categorical Units Reflecting Event History into Event Specific Disutility Scores Applicable to Diabetes Decision Models. Value Heal [Internet]. 2018;21:S223. Available from: https://doi.org/10.1016/j.jval.2018.04.1506

15. Diário da República. Portaria 207/2017, de 11 de Julho [cited 2022 22 Jun]. Available from: https://dre.tretas.org/dre/3025134/portaria-207-2017-de-11-de-julho. 2017.

16. Infomed. Inframed 2022 [Available from: https://extranet.infarmed.pt/INFOMED-fo/index.xhtml.

17. WHO Collaborating Centre for Drug Statistics Research. ATC/DDD Index 2022 [Available from: https://www.whocc.no/atc_ddd_index/.

18. Hoskins N, Tikkanen CK, Pedersen-Bjergaard U. The economic impact of insulin-related hypoglycemia in Denmark: an analysis using the Local Impact of Hypoglycemia Tool. J Med Econ. 2017;20:363–70.

19. Jakubczyk M, Lipka I, Pawęska J, Niewada M, Rdzanek E, Zaletel J, et al. Cost of severe hypoglycaemia in nine European countries. J Med Econ. 2016;19:973–82.

20. Gouveia M BM, Pinheiro L, Costa J e Vaz, Carneiro A . Custo e carga da doença atribuível à diabetes em Portugal: alguns resultados preliminares. Congresso Português de Diabetes, Vilamoura, Março 2010.

21. Barendregt JJ, Van Oortmarssen GJ, Vos T, Murray CJL. A generic model for the assessment of disease epidemiology: the computational basis of DisMod II. Popul Health Metr. 2003;1:4.

22. Administração Central do Sistema de Saúde. Termos de Referência para contratualização de cuidados de saúde no SNS para 2022.

23. International Agency for Research on Cancer. Estimated number of new cases of cancer in 2020, World, both sexes, all ages 2022. Cancer Today [Internet]. [cited 2023 Jun 23]. Available from: https://gco.iarc.fr/today/online-analysis-table?v=2020&mode=cancer&mode_population=continents&population=900&populations=900&key=asr&sex=0&cancer=39&type=0&statistic=5&prevalence=0&population_group=0&ages_group%5B%5D=0&ages_group%5B%5D=17&group_cancer=1&i

24. Costa J, Alarcão J, Amaral‐Silva A, Araújo F, Ascenção R, Caldeira D, et al. Os custos da aterosclerose em Portugal. Rev Port Cardiol [Internet]. 2021;40:409–19. Available from: https://www.revportcardiol.org/pt-os-custos-da-aterosclerose-em-articulo-S0870255120304601

25. Wolf PA, D’Agostino RB, Belanger AJ, Kannel WB. Probability of stroke: a risk profile from the Framingham Study. Stroke. 1991;22:312–8.

26. Diário da República. Despacho N^o^2482 2019 - Cuidados respiratórios domiciliarios 2019 [Available from: https://dre.pt/dre/detalhe/despacho/2482-2019-120847122.

27. Servico Nacional de Saude. Circular Normativa 2021 [Available from: https://www.acss.min-saude.pt/wp-content/uploads/2021/03/Circular_Normativa_3_2021.pdf.

28. Perelman J, Soares M, Mateus C, Duarte A, Faria R, Ferreira L, Saramago P V, P, Furtado C, Caldeira S, Teixeira MC SM. Methodological Guidelines for Economic Evaluation Studies. INFARMED - National Authority of Medicines and Health Products, I.P., Lisbon. Available online at www.infarmed.pt. 2019.

29. Sociedade Portuguesa para o Estudo da Obesidade (SPEO). Tratamento não cirúrgico da Obesidade do Adulto. 2018;1–93.

30. Direção-Geral da Saúde. Obesidade: Otimização da abordagem terapêutica no Serviço Nacional de Saúde. Programa Nac. para a Promoção da Aliment. Saudável. 2017.

31. Angrisani L, Santonicola A, Iovino P, Formisano G, Buchwald H, Scopinaro N. Bariatric Surgery Worldwide 2013. Obes Surg. 2015;25:1822–32.

32. OECD. Population data 2021 [Available from: https://data.oecd.org/pop/population.htm.

33. Trends in adult body-mass index in 200 countries from 1975 to 2014: a pooled analysis of 1698 population-based measurement studies with 19·2 million participants. Lancet (London, England). 2016;387:1377–96.

34. Sjöström L. Review of the key results from the Swedish Obese Subjects (SOS) trial - a prospective controlled intervention study of bariatric surgery. J Intern Med. 2013;273:219–34.

35. Holmes M. Literature review for evidence to populate the Novo obesity model - Final report. Unpublished. 2017.

36. Cederholm J, Eeg-Olofsson K, Eliasson B, Zethelius B, Nilsson PM, Gudbjörnsdottir S. Risk prediction of cardiovascular disease in type 2 diabetes: a risk equation from the Swedish National Diabetes Register. Diabetes Care. 2008;31:2038–43.

37. Hippisley-Cox J, Coupland C, Brindle P. Development and validation of QRISK3 risk prediction algorithms to estimate future risk of cardiovascular disease: prospective cohort study. BMJ. 2017;357:j2099.

38. Young T, Shahar E, Nieto FJ, Redline S, Newman AB, Gottlieb DJ, et al. Predictors of sleep-disordered breathing in community-dwelling adults: the Sleep Heart Health Study. Arch Intern Med. 2002;162:893–900.

39. Wendelboe AM, Hegmann KT, Biggs JJ, Cox CM, Portmann AJ, Gildea JH, et al. Relationships between body mass indices and surgical replacements of knee and hip joints. Am J Prev Med. 2003;25:290–5.

40. Adams KF, Leitzmann MF, Albanes D, Kipnis V, Mouw T, Hollenbeck A, et al. Body mass and colorectal cancer risk in the NIH-AARP cohort. Am J Epidemiol. 2007;166:36–45.

41. Renehan AG, Tyson M, Egger M, Heller RF, Zwahlen M. Body-mass index and incidence of cancer: a systematic review and meta-analysis of prospective observational studies. Lancet (London, England). 2008;371:569–78.

42. Renehan AG, Zwahlen M, Egger M. Adiposity and cancer risk: new mechanistic insights from epidemiology. Nat Rev Cancer. 2015;15:484–98.

43. Lopes S, Johansen P, Lamotte M, McEwan P, Olivieri A-V, Foos V. External Validation of the Core Obesity Model to Assess the Cost-Effectiveness of Weight Management Interventions. Pharmacoeconomics. 2020;38:1123–33.

44. Lopes S, Meincke HH, Lamotte M, Olivieri A-V, Lean MEJ. A novel decision model to predict the impact of weight management interventions: The Core Obesity Model. Obes Sci Pract. 2021;7:269–80.
